# Implementing PTSD interventions for hospital nurses and physicians during COVID-19: A scoping review

**DOI:** 10.1186/s13690-025-01706-0

**Published:** 2025-10-02

**Authors:** Deliah Katzmarzyk, Daniela Holle, Martina Roes

**Affiliations:** 1 Faculty of Health, School of Nursing Science, Witten/Herdecke University, Witten, Germany; 2 Hannover Medical School, Institute for Epidemiology, Social Medicine and Health Systems Research, Hannover, Germany; 3 Bochum University of Applied Sciences, Department of Nursing-, Midwifery, -and Therapeutic Sciences, Bochum, Germany; 4 German Center of Neurodegenerative Diseases (DZNE), Witten, Germany

**Keywords:** Implementation science, Stress disorders, Post-traumatic, Nurses, Physicians, Hospitals, COVID-19

## Abstract

**Background:**

Nurses and physicians in hospitals are particularly affected by the impacts of the COVID-19 pandemic as shown in the high prevalence of post-traumatic stress disorder (PTSD). To handle the urgent and high demand for psychological support, PTSD-related interventions had to be applied rapidly. Thus, interventions that were already evidence-based were adapted to pandemic conditions, or new interventions were developed. To implement these interventions sustainably, and be prepared for future disease outbreaks, we need to identify which strategies are necessary for the successful implementation. From this perspective, four years after the COVID-19 outbreak, we address the following:

*What are the [1] interventions that address symptoms of post-traumatic stress disorder in hospital-based nurses and physicians during the COVID-19 pandemic? What are the [2] implementation strategies for the identified interventions?*

**Methods:**

We used a scoping review approach and conducted a literature search from February to April 2023 in PubMed, PsychINFO and CINHAL. Primary studies (protocols) and concept papers focused on PTSD-related interventions for nurses and physicians and their implementation in hospitals during the COVID-19 pandemic, and published between 2020 and 2023 were included. Data extraction and analysis were performed in MaxQDA using deductive content analysis based on the (a) template for intervention description and replication (TIDieR) and the (b) Expert recommendations for implementing change (ERIC) framework.

**Results:**

A total of 16 interventions were adapted or developed world wide during the COVID-19 pandemic between 2020 and 2023. Evidence of effectiveness exist in only six of the 16 interventions. Most of them were designed using digital approaches and were primarly delivered through iterative implementation cycles, whereas the implementation of face-to-face interventions focused on interactions with various stakeholders.

**Conclusion:**

Our findings can be used to support the implementation of PTSD-related interventions for nurses and physicians in hospitals under pandemic conditions. Future research should focus on evaluating the effectiveness of these interventions and identifying strategies for a beneficial and sustainable implementation.

**Supplementary Information:**

The online version contains supplementary material available at 10.1186/s13690-025-01706-0.

**Table:** 

Text box 1. Contributions to literature
• We found limited evidence for a few interventions to improve symptoms of post-traumatic stress disorder among hospital-based nurses and physicians that were developed or adapted for COVID-19 pandemic conditions.
• Despite the lack of implementation studies, we found that two different methods of delivering the interventions were identified: face-to-face and digital. The analyzed implementation strategies highlight differences in the implementation of these interventions.
• These findings extend implementation science and practice in the field of mental health during disease outbreaks, by providing knowledge of PTSD-interventions and implementation strategies to use them in an effective and sustainable way.

## Introduction

During the global outbreak of the coronavirus disease 2019 (COVID-19), the number of hospitalized COVID-19 patients increased [1]. At the same time the psychological burden for health care workers (HCW), particularly nurses and physicians, increased substantially [2]. Several studies investigated the prevalence of various psychological issues during the COVID-19 pandemic on HCW. The prevalence of depression, anxiety, insomnia, stress, and PTSD was higher than other mental disorders, particulary among nurses and physicians [3]. A meta-analysis shows that nurses and female HCWs experienced the highest burden of PTSD symptoms compared with the public [2]. Additionally, nurses were the most affected professional group among HCWs, with symptoms of depression, or anxiety still present in 2021 after the pandemic, compared with their occurrence among physicians [4].

When the awareness about the high psychological burden of nurses and physicians during the COVID-19 pandemic grew, concerns about severe long-term consequences for the entire health care sector increased [5]. In particular symptoms of PTSD such as flashbacks or intrusive thoughts [6] might have a longer-lasting effect on those professional groups [5]. Therefore, researchers emphasize an urgent need for interventions to improve the mental health of this professional group and call for action for public health agencies and institutions, such as hospitals [7–9].

To provide psychological support for nurses and physicians experiencing symptoms of PTSD as quickly as possible [10], researchers recognized the translational potential of already developed interventions, such as cognitive behavioral therapy (CBT) [4]. Since social distancing measures were required during the pandemic, in-person interventions were adapted, for example, using digital modalities to enhance the accessibility [5–7].

To implement those modified or newly designed PTSD-interventions for hospital-based nurses and physicians beneficially and sustainably, appropriate strategies and methods are required [11]. According to the literature, these strategies are defined as “methods or techniques used to enhance the adoption, implementation, and sustainability” [12] of interventions.

One possible approach is provided by the ERIC-framework developed by Powell et al. [12], which consists of 73 validated and clearly defined characteristic strategies. Graham et al. [13] theoretically adapted these strategies for implementing digital mental health interventions (DMHI). It is uncertain if these conceptually designed strategies are effective. Furthermore, they are not conceptualized for a specific implementation context such as a hospital, nor a specific DMHI [13]. In addition to a specific PTSD-intervention, a clearly defined implementation context is also required to provide tailored strategies [14].

Existing reviews focus on mapping PTSD-related interventions for nurses working in a hospital [15] or on investigating the effectiveness of those interventions through systematic review and meta-analysis [16]. To date, no review exists, that maps PTSD-related interventions and identifies strategies, that could be applied to implement these interventions for hospital-based nurses and physicians during the COVID-19 pandemic.

In response to this gap, our study seeks to map PTSD-related interventions and explore implementation strategies that address nurses and physicians working in an acute hospital setting. Our scoping review was guided by the following central research questions:

*What are the [1] interventions that address symptoms of post-traumatic stress disorder in hospital-based nurses and physicians during the COVID-19 pandemic? What are the [2] implementation strategies for the identified interventions?*

## Methods

Since we aim to explore and map the existing PTSD-related interventions for nurses and physicians working in a hospital, analyze implementation strategies, and identify research gaps in the implementation of those interventions, we conducted a scoping review [17]. This was conceptualized based on the methodology of the Joanna Briggs Institute (JBI) and the approach of Peters et al. [17]. For consistency in reporting, we used the PRISMA Extension for Scoping Reviews (PRISMA-ScR) [18], which is presented in additional file 1.

### Selection criteria and sources of information

We operationalized our research question, using the PCC-elements (**P**opulation, **C**oncept, and **C**ontext) framework [17] and defined our selection criteria (see Table 1).

**Table 1:** In- and exclusion criteria according to the PCC elements

Criteria	Inclusion	Exclusion
Population	Nurses and physicians with symptoms of post-traumatic stress disorder (PTSD)	Other professions (e.g., Community Health Nurses, physiotherapist, respiratory therapist)
Concept	Interventions related to symptoms of PTSD, and strategies to implement these interventions	Non-PTSD related interventions
Context	Acute somatic hospital setting during the COVID-19 pandemic	Specialized clinics such as mental/psychiatric hospital
Types of evidence sources	Evaluation and implementation studies, study protocols, feasibility studies, concept articles	Reviews
Other	Language: German and English Publication time: 2020—2023	Published before 2020 and after 2023

As population we defined nurses and physicians showing symptoms of PTSD. To specify these symptoms, we applied the definition from the International Statistical Classification of Diseases and Related Health Problems 10th Revision (ICD-10) – Chapter V for PTSD [6]. Our concepts include PTSD-related interventions and strategies to implement these interventions. At least we defined the context as the acute somatic hospital setting and the COVID-19 pandemic period from 2020 to 2023. We selected this time period to specifically capture how psychological support was provided for hospital-based nurses and physicians right within the COVID-19 period. This inclusion criteria is justified by the well-documented increase in mental health problems and extraordinary demands placed on this professional group during this global health crisis [5, 7].

Beyond, we included all reviews that met the eligibility criteria to identify studies throughbackward citation screening, although the reviews themselves were not part of the analysis.

Articles were excluded if they described non-PTSD-related interventions and addressed nurses or physicians working in other contexts, such as mental or psychiatric hospitals.

We conducted the literature search in MEDLINE via PubMed, PsychINFO and CINHAL via EBSCO between February and April 2023. A research protocol with detailed information about the literature search is available in additional file 2.

### Search and selection of source of evidence

We used the Ref Hunter in web format by Nordhausen and Hirt [19] as a general guide for conducting and reporting a transparent and comprehensive systematic literature search.

Before the development of all the search strings, one researcher (DK) conducted an initial limited search in MEDLINE, PsychINFO, CINHAL and Google Scholar to identify synonyms and keywords of each search term. The search strings were developed by one researcher (DK) and independently verified by two other researchers (DH, MR) via the Peer Review of Electronic Search Strategies (PRESS) [20]. First, we constructed a search string for MEDLINE and modified it for PsychINFO and CINAHL according to the specific functions of each database.

The developed search strings were deposited online, with weekly alerts for new articles.

To enhance the systematic research, we used subsequent supplementary search options following Cooper’s et al.'s [21] recommendations. We screened the reference lists of included articles for relevant publications and searched in Google Scholar using the forward citation screening. We also performed a trial register and a hand search via Google Scholar.

After that, we transferred the identified articles to EndNote 20.5 to exclude all duplicates. The remaining articles were uploaded to the online tool Rayyan [22] for literature screening. The title-abstract and full-text screening was performed in two iterations by DK. Furthermore, two researchers (DH, MR) independently screened four randomly selected articles to strengthen the quality of our scoping review. Any conflicts were discussed by DK, DH, and MR until a consensus was reached. We used the PRISMA flowchart [23] for presenting our literature search.

### Data extraction and analysis

We extracted and analyzed the data using MaxQDA version 2022 in two distinct steps, aligning with the objectives: to [1] explore and map the interventions and to [2] present implementation strategies in the implementation of PTSD-related interventions.

First, we extracted general information to delineate the characteristics of the included studies, such as publication year, the intervention, study period, and design, as well as the type of article. To extract the information about the interventions, we used the template for intervention description and replication (TIDieR) [24]. We continued the data extraction along the 12 items of the TIDieR. Two iterations were performed by DK. Additionally, two other researchers (DH, MR) extracted data from randomly selected articles independently.

We present a brief overview of the results in a comprehensive table (see Table 3) and in the results section along the items of the TIDieR [24].

Second, we performed a deductive content analysis using the terminology of the implementation strategies by Powell’s et al. [12]. The analysis was performed by one researcher (DK) in two iterations. After the first iteration, an exchange with another researcher (MR) was conducted to discuss conflicts and reduce bias. To gain a better understanding of how we analyzed the implementation strategies, we provided some examples of coding in Table 2.

**Table 2:** Examples of the coding of implementation strategies within their respective thematic clusters

Identified thematic clusters [25] and implementation strategies [12]	Examples of coding
*Use evaluative iterative strategies*	
Audit and provide feedback	*‘Visitors have been asked *via* electronic mail to tell us what they want from the Bubble, how it helps them, and how it could do better, in what we might describe as a free-text qualitative survey.’* [26]
Obtain and use patients/consumers and family feedback	*‘Additionally, individual telephone discussions were held with the 5 strategic role-holder PPI participants (3 nurses, 1 physiotherapist, 1 medical doctor) who provided further comment and suggestion around elements of the package content relating directly to COVID-19 and psychological wellbeing.’* [27]
Conduct local need assessment	*‘Representatives from the Steering Committee meet with departmental or unit leadership to learn about their unique needs and stressors and explain the proposed program. This is followed quickly by “all-hands” launch meetings with faculty and frontline personnel (conducted remotely *via* teleconferencing), to ensure horizontal spread and acceptance of the program.’* [28]
*Change infrastructure*	
Change service sites	*‘The “My Health Too” website was initially developed by a team of developers, designers, illustrators, and videographers during a Hacking Health Camp event— […]’* [29]
*Develop stakeholder interrelationships*	
Identify and prepare champions	*‘Once matched, each site is asked to identify at least one site leader; intervention sites also identified site champions (at least one champion per every 50 HCW planned to receive the intervention), […]’* [30]
*Train and educate stakeholders*	
Conduct educational meetings	*‚Healthcare workers and healthcare students were recruited over 3 days *via* professional networks and provided with a link to Version 1.0 of the digital package.’* [27]
Conduct ongoing training	*‚In stage 2, the process involves concurrent training for remote PFA providers and promotion of the service *via* the hospital’s website, social media, and posters.’* [31]
Use train-the-trainer strategies	*‘These will be delivered over 1-day face-to-face simulation training course (7 h) and two follow-up practice supervision sessions (1 h each); with a focus on improving the trainers’ knowledge, skills, and self-efficacy related to support people in acute stress.’* [32]
*Engage consumers*	
Involve patients/consumers and family members	*‘Throughout this process, stakeholder participation in its development was achieved through: (A) conducting individual interviews (n* = *15) of healthcare staff to capture their perceived needs (e.g., case range of application context) and training preferences; […]’* [32]
*Adapt and tailor to context*	
Tailor strategies	*‘This approach was undertaken to ensure the intervention is scalable and can also be implemented 7 when time is sparse and personal contacts are restricted due to risk of contagion.’* [33]
Promote adaptability	*‘Online delivery was essential given ongoing pandemic-related restrictions to in person services; […]’* [34]
*Provide interactive assistance*	
Provide clinical supervision	*‘The therapists received regular and daily 1 h group supervision by EMDR EUROPE Accredited Consultants and worked in the presence of a supervisor.’* [35]

For presenting our results, we created a comprehensive in which the analyzed implementation strategy are presented for each respective intervention (see Table 4). Additionally, we categorized the strategies based on the intervention format (digital or face-to-face).

### Critical appraisal of individual sources of evidence

As this article meets the requirements of a scoping review, no critical appraisal of the included studies was conducted.

## Results

**Fig. 1:**
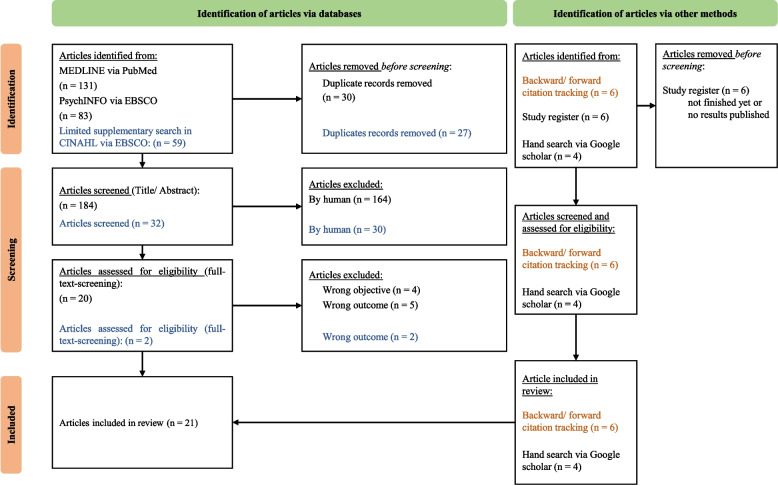
PRISMA flowchart [23]

The systematic literature search in PubMed, PsychINFO, and CINHAL resulted in 273 records, which were reduced to 216 after removing duplicates. These were screened in the following title-abstract screening, where 22 articles were identified for the full-text screening. In addition, five ongoing trials were identified from trial registries. These studies were excluded because they were ongoing trials with no published study protocol or results. Additionally, six articles were eligible from backward and forward citation screening and four from a hand search via Google Scholar. In the end, a total of 21 studies were included for data extraction and analysis (see Fig. 1).

### Study characteristics

The majority of the studies were conducted in Europe (*N* = 11) and North America (*N* = 7) in the early phase of the COVID-19 pandemic (2020–2021). They were designed as empirical studies with a quantitative approach (*N* = 14). Most of the studies were published in 2022 (*N* = 5). Six studies were planned between 2020 and 2023 and published as study protocols. Furthermore, one study was published as a concept paper. The study characteristics are available in Table 3.

**Table 3:** Characteristics of the included studies (*N* = 21)

Publication	Year	Location	Intervention	Evidence level	Study	Study period	Study design	Type of article
Albott, C. et al	2020	Minesota, USA	Battle Buddies—Psychological Resilience intervention based on *Anticipate-Plan-Deter (APD)* [face-to-face]	‘Evidence-informed’	Battle Buddies': Rapid Deployment of a Psychological Resilience Intervention for Health Care Workers During the COVID-19 Pandemic	Not mentioned	Not mentioned	Concept paper
Blake, H. et al	2020	United Kingdom	Digital learning package [digital]	not categorizable	Mitigating the Psychological Impact of COVID-19 on Healthcare Workers: A Digital Learning Package	February-April 2020	Based on a three-step process, including public involvement activities, content and technical development with iterative peer review, delivery, and evaluation	Empirical paper
Bureau, R. et al	2021	Strasbourg, France	My Health Too based on *Cognitive behavioral Therapy (CBT), Psychoeducation by Lazarus and Folkman´s transactional stress model* [digital]	‘Evidence-informed’	My Health Too: Investigating the Feasibility and the Acceptability of an Internet-Based Cognitive-Behavioral Therapy Program Developed for Healthcare Workers	May—September 2021	Feasibility study with using an internet survey and individual interviews	Empirical paper
Dong, L. et al	2022	California USA	Stress First Aid (SFA) based on *Stress continuum and Psychological First Aid (PFA)* [digital]	‘Evidence-informed’	Mental and Physical Well-Being of Frontline Health Care Workers During the Coronavirus Disease 2019 (COVID-19) (COVER-HCW)	March 2021—November 2023	A mixed-methods approach, includes a quantitative component designed as a cluster-randomized-controlled trial (cRCT) with three arms and a qualitative component designed as a complementary descriptive study	Study protocol
Dumarkaite, A. et al	2023	Lithuania	Internet-delivered stress recovery intervention (FOREST) based on *Cognitive-behavioral therapy (CBT) and mindfulness* [digital]	Evidence-based	Stress Recovery Program FOREST for Healthcare Staff (FOREST)	April 2021 -December 2022	A randomized-controlled trial (RCT) parallel groups waiting list design with three measurement points	Empirical paper
Fiol-DeRoque, M. et al	2021	Palma de Mallorca, Spain	PsyCovidApp based on *Cognitive behavioral Therapy (CBT) and mindfulness* [digital]	‘Evidence-informed’	A Mobile Phone-Based Intervention to Reduce Mental Health Problems in Health Care Workers During the COVID-19 Pandemic (PsyCovidApp)	May 2020 -August 2020	A randomized-controlled-trial (RCT)	Empirical paper
Fogliato, E. et al	2022	Rome, Italy	Eye Movement Desensitization and Preprocessing Therapy (EMDR) [face-to-face]	Evidence-based	Promoting Mental Health in Healthcare Workers in Hospitals Through Psychological Group Support With Eye Movement Desensitization and Reprocessing During COVID-19 Pandemic (HOPE)	March 2020—June 2021	Observational study	Empirical paper
Hannig, C. et al	2021	Hamburg, Germany	Hamburger concept based *on peer approach* [face-to-face]	Not categorizable	Stress and Trauma Prevention for health-care workers	Not mentioned	For the evaluation of peer education, a questionnaire was used based on the four-levels model by Kirkpatrick (2006). To evaluate the acceptance of the education, a questionnaire with a five-level Likert-scale was used. Also, open-end questions were performed to evaluate the positive or negative experiences, as well as further suggestions. Additionally, pre-, and post- assessment of personal security level according to the general commerce as peers was conducted	Empirical paper
Jovarauskaite, L. et al	2021	Lithuania	Internet-delivered stress recovery intervention (FOREST) based on *Cognitive-behavioral therapy (CBT) and mindfulness* [digital]	Evidence-based	Stress Recovery Program FOREST for Healthcare Staff (FOREST)	April 2021—December 2022	A randomized-controlled trial (RCT) parallel groups waiting list design with three measurement points	Study protocol
Kanellopoulos, D. et al	2021	New York City, USA	CopeNYP based on *Psychological First Aid (PFA)* [digital]	Evidence-based	The CopeNYP program: A model for brief treatment of psychological	March 2020—April 2021	Initial uncontrolled trial evaluation	Empirical paper
Lefevre, H. et al	2021	Paris, France	The Port Royal Bubble (La Bulle de Port Royal) [face-to-face]	Not categorizable	The Bulle: Support and Prevention of Psychological Decompensation of Health Care Workers During the Trauma of the COVID-19 Epidemic	Not mentioned	Evaluation of the frequency of use within the different professions using a quantitative approach. Additionally, using electronic mail to collect data regarding the expectation of the program, way of helping and further suggestions	Empirical paper
Mellins, C. et al	2020	USA	CopeColumbia based on *Cognitive-behavioral therapy (CBT), Acceptance and Commitment Therapy (ACT)* [digital]	‘Evidence-informed’	Supporting the well-being of health care providers during the COVID-19 pandemic: The CopeColumbia response	March—June 2020	Evaluation of themes raised by participants, facilitator intervention, and the perceives impact of the program. Therefore, 1) weekly peer supervision discussions provided qualitative information and clinical expert consensus, and 2) an anonymous, confidential, and voluntary post-group brief Qualtrics survey (≤ 2 min) link was emailed to participants who volunteered their contact information	Empirical paper
Morina, N. et al	2021	Switzerland	RECHARGE based on *Psychoedcuation* [digital]	‘Evidence-informed’	RECHARGE: A Brief Psychological Intervention to Build Resilience in Healthcare Workers During COVID-19	August 2020—June 2021	A randomized-controlled trial (RCT)	Study protocol
Serrano-Ripoll, M. et al	2021	Spain	PsyCovidApp based on *Cognitive behavioral Therapy (CBT) and mindfulness* [digital]	‘Evidence-informed’	Mobile Phone Based Intervention to Protect Mental Health in Healthcare Workers at Frontline Against COVID-19 (PsyCovidApp)	May 2020 –August 2020	A randomized-controlled-trial (RCT)	Study protocol
Sagaltici, E. et al	2022	Turkey	Online format of the *Recent Event and Eye Movement Desensitization* (EMDR) [digital]	Evidence-based	Recent Traumatic Episode Protocol EMDR Applied Online for COVID-19-Related Symptoms of Turkish Health Care Workers Diagnosed with COVID-19 Related PTSD	September 2020—December 2020	A pilot study with investigation of the effect of the intervention	Empirical paper
Solomonov, N. et al	2022	New York City, USA	CopeNYP based on *Psychological First Aid (PFA)* [digital]	Evidence-based	CopeNYP: a brief remote psychological intervention reduces health	March 2020—April 2021	Evaluation of the programs’ efficacy in reducing depression and anxiety symptoms using the established questionnaires	Empirical paper
Sulaiman, A. et al	2020	Malaysia	Remote-PFA based on *Psychological First Aid* [digital]	‘Evidence-informed’	Development of a Remote Psychological First AId Protocol for Healthcare Workers Following the COVID-19 Pandemic in a University Teaching Hospital	Not mentioned	Stepwise implementation of the intervention within a healthcare system based in the ‘goal setting’ approach with using quantitative and qualitative for the evaluation	Empirical paper
Trottier, K. et al	2021	Canada	Recovering from Extreme Stressors Trough Online Resources and E-health (RESTORE) based on *Cognitive Processing Therapy (CPT)* [digital]	‘Evidence-informed’	RESTORE: an online intervention to improve mental health symptoms associated with COVID-19-related traumatic and extreme stressors	March 2021 – November 2021	Using self-reported measures at baseline, mid-intervention, end-of-intervention, and at 1-month follow-up within the module assessments to assess the condition of each participant. Additionally, qualitative interviews administered after the intervention period. Changes in mental health symptoms will be examined to evaluate preliminary efficacy. Feasibility will be assessed through recruitment, retention, and adherence rated, as well as additional analytics and participants feedback	Empirical paper
Trottier, K. et al	2022	Toronto Canada	Recovering from Extreme Stressors Trough Online Resources and E-health (RESTORE) based on *Cognitive Processing Therapy (CPT)* [digital]	‘Evidence-informed’	RESTORE: an online intervention to improve mental health symptoms associated with COVID-19-related traumatic and extreme stressors	March 2021 – November 2021	An uncontrolled trial	Empirical paper
Wang, L. et al	2020	China	Preparing ME based on *Psychological First Aid (PFA) and the RAPID-Model* [face-to-face]	‘Evidence-informed’	Evaluating a Psychological First Aid Training Intervention (Preparing Me) to Support the Mental Health and Wellbeing of Chinese Healthcare Workers During Healthcare Emergencies ('Preparing Me'-project)	Not mentioned	A two arm, feasibility randomized controlled trial	Study protocol
Weiner, L. et al	2020	Strasbourg France	My Health Too based on *Cognitive behavioral Therapy (CBT), Psychoeducation by Lazarus and Folkman´s transactional stress model* [digital]	‘Evidence-informed’	REduction of Stress (REST)	May—September 2021	A six-site, prospective, randomized, open and parallel group-controlled study with two arms	Study protocol

This table also shows the level of evidence presented for the interventions, and in the box for describing the intervention, the mode of delivery—face-to-face or digital – is shown in brackets

### Intervention characteristics

In total sixteen interventions for nurses and physicians were explored. Nine of these are categorized as ‘evidence-informed’, which means that these PTSD-interventions are modified or new developed during the COVID-19, but without an existing proof of effectiveness. Of the sixteen interventions, four were identified as evidence-based, as their effectiveness had been evaluated in dedicated studies.

Further, five interventions were delivered face-to-face, and eleven were digital.

#### Aims of the intervention and their theoretical approaches

All studies reported about a rationale and aim for developing an intervention, such as being aware of the need for self-care [26, 28], strengthening of resilience [26] or support for psychological well-being [27, 36, 37] of nurses and physicians. Additionally, PTSD-related interventions have the potential to mitigate the long-term mental health impacts on nurses and physicians [27, 30], and to improve symptoms of PTSD or other related disorders [34, 38]. Fogliato et al. [39] reported about a more specific intervention-based goals, like for the EMDR to *“[…] restore a natural way of processing the information in the memory to achieve an adaptive resolution through the creation of new, more functional connection.”* [39].

Most of the interventions are theory-driven, drawing on a model such as the Anticipate-Plan-Deter (APD) model [28], the psychoeducational model, based on Lazarus and Folkman’s transactional stress model [29, 40], the stress continuum model [30], the Adaptive Information Processing (AIP) model [39] or the RAPID model [41]. Some interventions did not use an underlying theoretical approach [27, 36, 37].

#### Form and modality of intervention use

Many of the interventions included some form of peer support, for example, support provided by other staff, colleagues or team members. This component was used in various ways, for example, as the main component of the intervention [26–28, 42, 43] or as an additional component of an evidence-based intervention, such as in PFA [30] or CBT [33].

Most of the interventions were classified as ‘evidence-informed’ consists of CBT, PFA, or other components [11, 28–31, 33, 34, 38, 40, 41, 43, 44]. In contrast, four interventions are considered evidence-based and include components such as EMDR [35, 39], CBT [45, 46] or PFA [36, 37].

Other interventions are designed by integrating additional components, like a telephone-hotline or a supervision [29, 36, 37, 40]. For at least three interventions, it was not possible to determine whether they consist of evidence-based or evidence-informed components [26, 27, 42].

Most of the intervention were applied using a digital modality [27, 29, 31, 33–38, 40, 43–47] and by trained professionals with expertise, such as in CBT [29, 40] or CBT and mindfulness [45, 46] or PFA [35–37] or EMDR [35].

#### The duration of the interventions

Depending on the modalities used, the sessions and modules of the interventions were not fixed to a specific duration, allowing flexible use of the intervention [27, 28, 45, 46]. The developers of the interventions provided an estimated duration of each session [29, 40]. They vary from 90 min [32, 39] to 30–60 min [33, 34, 38] to 20 min [43].

Some interventions are designed as programs so that developers are able to estimate the duration of the entire intervention, which varies from one day (eight hours) [27, 41] to eight weeks [29, 34, 38, 40]. Some studies do not provide information about the duration [30, 31, 36, 37, 42, 44, 47].

#### Adaptations of the intervention

None of the applied interventions are tailored to a specific professional group, although some authors report that the interventions are designed as need-based [28, 36, 37].

In addition, most of the studies report on unmodified interventions[29, 34, 35, 38, 40, 45, 46]. These can be understood as interventions described in a study protocol and using in a RCT without any reported adaptations. In contrast, one primary study [27] reports on two versions of an intervention but provides a detailed description only of the initial design. At least, several studies do not provide any information about adaptations of the interventions [26, 30, 31, 43].

### Implementation strategies

PTSD-related interventions for nurses and physicians working in hospitals during the COVID-19 pandemic are delivered using a range of modalities, including face-to-face and digital formats.

However, none of the analyzed studies explicitly report about applied implementation strategies or the evaluation of such strategies in terms of implementation outcomes like adoption, acceptance or feasibility [48]. Nevertheless, some familiar terms align with the discrete implementation strategies provided by Powell et al. [12] could be analyzed. Table 4 provides an overview of analyzed implementation strategies for each identified PTSD-intervention. The strategies used to implement PTSD-related interventions depend on modality used.

**Table 4:** Implementation strategies for each identified PTSD-intervention, classified in digital or face-to-face intervention

Implementation strategies [12] and thematic cluster [25]	Digital intervention																	Face to face intervention						
	Digital learning package [27]	My Health Too [29, 40]			Stress First Aid (SFA) [30]		FOREST [45, 46]		PsychCovid-App [44, 47]	CopeNYP [36, 37]	CopeColumbia [43]		RECHARGE [33]	EMDR [35]		Remote PFA [31]	RESTORE [34, 38]	Battle Buddies [28]	EMDR [39]	Hamburger concept [42]	The port Royal Bubble [40]			Preparing ME [44]
Use evaluative and iterative strategies																								
Assess for readiness and identify barriers and facilitators	×	×																						
Audit and provide feedback	×	×					×		×		×					×	×	×			×			×
Purposefully reexamine the implementation																×								
Develop and implement tools for quality monitoring		×														×								
Develop a formal implementation blueprint					×					×						×								
Conduct local need assessment	×								×							×		×						×
Stage implementation scale up	×															×								
Obtain and use patients/consumers and family feedback	×						×				×					×	×				×			
Conduct cyclical small tests of change	×																							
Provide interactive assistance																								
Provide clinical supervision							×			×	×		×	×				×						×
Adapt and tailor to context																								
Tailor strategies										×	×		×			×				×				
Promote adaptability											×		×			×	×	×		×	×			×
Develop stakeholder interrelationships																								
Identify and prepare champions	×		×							×						×		×		×			×	
Organize clinician implementation team meetings										×								×		×				
Inform local opinion leaders								×				×												
Use advisory boards and workgroups																		×						
Train and educate stakeholders																								
Conduct ongoing training						×										×								
Develop educational materials						×																	×	
Distribute educational materials																							×	
Use train-the-trainer strategies						×							×							×			×	
Conduct educational meetings	×									×								×		×			×	
Conduct educational outreach visits						×																×		
Create a learning collaborative																				×				
Engage consumers																								
Involve patients/consumers and family members	×															×		×					×	
Intervene with patients/consumers to enhance uptake and adherence	×															×								
Increase demand																				×				
Use mass media																×								
Change infrastructure																								
Change physical structure and equipment																			×			×		
Change service sites	×			×			×	×				×	×		×	×	×							

In total 99 times was an implementation strategy coded in the included studies. Of them, 27 are identified as implementations of face-to-face interventions and 42 as digital interventions. Across both face-to-face and digital modalities, the most frequent applied strategy is ‘audit and provide feedback’ [25] (*N* = 10), which involves implementing interventions through iterative and evaluative cycles [12].

#### Strategies used for face-to-face interventions

The most common analyzed thematic cluster of strategies is ‘develop stakeholder interrelationships’ [25], in which a participatory approach is used to identify and prepare individuals as peers, potentially facilitating the implementation of PTSD-related interventions [12, 28, 41, 42]. For instance, the peers in Hannig et al.’s [42] intervention create their own methods to apply the ‘Hamburger concept’ [42] within their teams in the hospital. This intervention incorporates components of primary, secondary, and tertiary prevention, such as educational resources for managing stressful situations and psychological support or consultation, including screening of potential psychological issues [42].

Another implementation strategy applied is ‘promote adaptability’, which emphasizes the need for interventions to be tailored to the specific needs of the target group and designed to align with existing work structures, enabling nurses and physicians to use them effectively [12, 26, 28, 35, 41, 42]. For example, Fogliato et al. [39] and Lefevre et al. [26] describe the creation of dedicated physical spaces with separate rooms for peer socializing and the delivery of the exercise-based intervention.

Additionally, ‘train and educate stakeholders’ was identified, which involves designing and delivering training sessions for nurses, physicians, and other involved personnel [12, 28, 41, 42]. One such intervention “*Preparing ME”*, developed by Wang et al. [41], prepare individuals, who could function as ‘trainers’ [41]. These individuals receive instruction on how to use the intervention and implement it in group sessions or case-based simulations [41].

#### Strategies used for digital interventions

The most commonly analyzed thematic cluster of strategies is ‘use evaluative and iterative strategies’ [25], in which digital interventions are implemented through ongoing evaluative cycles [12]. The approach allows researchers to gather feedback from nurses and physicians – for example, in terms of technical problems during use or overall experience with the intervention [12, 34, 36–38, 43]. For instance, Sulaiman et al. [31] used a ‘goal-setting approach’ [31] involving hospital stakeholders, such as nurses, physicians, and the management, in iterative implementation cycles to receive feedback.

Another frequently coded strategy is ‘change service sites’ [12], which highlights the need to adapt digital interventions to changing circumstances, such as those experienced during the COVID-19 pandemic. Many digital interventions are delivered via online platform [27, 29, 34, 38, 40, 43, 45, 46] or a mobile applications [31, 44, 47]. For example, Morina et al. [33] designed the intervention *RECHARGE* using a video conferencing platform to deliver its content.

Additionally, the strategy ‘provide clinical supervision’ [12] is commonly used in implementing digital PTSD-related interventions. This strategy offers intervention providers the opportunity to participate in supervision sessions to share their experiences and perceptions during the implementation process [12, 34–38]. For instance, the intervention *FOREST* [45, 46] includes psychologists who offered supervision for sharing their experiences and overcome arising problems or challenges.

## Discussion

### PTSD-related interventions for nurses and physicians working in hospitals during the COVID-19 pandemic

Most interventions are categorized as ‘evidence-informed’ and are created in a digital modality during the COVID-19 pandemic. Given the necessity of rapidly deploying interventions to meet the acute psychological support needs of nurses and physicians [49], the evaluation of intervention effectiveness was often deprioritized. Evidence-based psychological interventions, such as CBT or EMDR, are originally delivered in person and for the general population before the pandemic. Social restrictions or individual concerns to limiting contracting the virus are reasons for adapting these interventions to pandemic conditions [50]. Witteveen et al. [50] reached a similar conclusion, noting that the use of in-person services declined between 2020 and 2021, while applying remote interventions increased. Particularly for HCWs, especially nurses and physicians a highly flexible and efficient use of interventions is crucial, since long and overly stressful shifts with increased psychological stress [51] lead to generally less efficient time use. Therefore, the 'evidence-informed' or evidence-based interventions identified in our scoping review were often designed as digital interventions to be compatible with the challenging conditions. To provide timely support, the use of a modified evidence-based intervention that can be applied under pandemic conditions represents an efficient and pragmatic approach. In general, given the dynamic nature of infections and the increasing burden on professionals, researchers emphasize the need for rapid development of interventions [28, 43, 49].

In addition, a significant number of studies were study protocols. These describe the intervention its intended delivery, but do not report on the actual implementation for nurses and physicians working in hospitals.

Between 2020 and 2023, a total of 13 interventions for PTSD symptoms have been made available to nurses and physicians and other HCWs worldwide. Given the geographical distribution of the studies, this is basically a poor result, considering the high prevalence of PTSD symptoms worldwide among HCWs three years after the beginning of the pandemic [3]. Before starting an implementation, the interventions had to be either adapted to or newly developed for the pandemic conditions to address the PTSD symptoms of HCWs. Consequently, these interventions had to be implemented rapidly, leaving little time or resources for conducting effectiveness studies. Overall, the identified 13 interventions represent an important step towards a timely response to the observed PTSD symptoms among nurses and physicians working in acute hospitals. Despite this, the effectiveness of the identified interventions reamins limited and heterogeneous. Studies investigating the effectiveness indicated positive effects on traumatization [39, 45] or, at a minimum, on anxiety [37].

In contrast, some studies concluded that the intervention was not effective in treating PTSD or traumatization [47]. However, intervention studies of these adaptive digital interventions are essential to demonstrate their actual effectiveness, additionally to the feedback already received from recipients. Empirical studies have examined the usefulness, practicability, or feasibility of interventions [34, 36, 37, 43]. In other studies, formative evaluation accompanied the development process via interviews and questionnaires [27, 42]. This indicates that stakeholders involved in the development and implementation process served as participants indata collection. This approach offered the possibility to identify the needs of HCWs for tailoring the intervention and for conducting a ‘step-by-step’ implementation with evaluative parts [52].

The study characteristics indicate that the included study protocols represent ongoing research in this area. Consequently, the effectiveness of developed interventions during the COVID-19 pandemic is expected to be evaluated in forthcoming intervention trials. Finally, the results of the intervention studies could be served as recommendations for decision makers in hospitals, as well as for their application beyond the context of the COVID-19 pandemic and the hospital setting [6].

### Strategies to implement PTSD-related interventions

Digital interventions were delivered by using evaluative and iterative methods, while the face-to-face interventions were applied with a participatory approach including stakeholders’ involvement. Strategies for implementing PTSD-related interventions are commonly used across both digital and face-to-face modalities: ‘audit and provide feedback’, ‘promoting the adaptability’, ‘providing clinical supervision’ and ‘changing service sites’ [12].

The most notable finding is that while the delivery of PTSD-related interventions was described, the specific strategies or methods used to implement these interventions were often not reported. Given that none of the included studies were explicitly designed as implementation studies, this finding is not surprising. However, implementation components could still be identified within the studies as implicit elements, even though they were not explicitly labeled or described as such—for example, using terminology provided by the ERIC framework [12]. An example of an implicit element were, the use of a ‘co. design’ with involving nurses and physicians in the implementation process as active participants [53]. By using those implicit elements, it was possible to identify and classify methods used in the delivery of PTSD-related interventions based on the ERIC framework [12].

*‘Audit and provide feedback’* [12] was the most frequent analyzed implementation strategy among PTSD-related interventions. Owing to the large number of empirical studies and study protocols, as well as that most digital interventions are ‘evidence-informed’, this result is not surprising. The studies apply or plan to employ feedback from involved stakeholders to evaluate the interventions’ delivery, but also the intervention itself in terms of utility, practicability, and feasibility. These results coincide with the strategies reported by Graham et al. [13]. The authors mention that need to evaluate both the implementation process and the intervention itself by receiving feedback from multiple perspectives, including hospital management as well as nurses and physicians – the primary users of the intervention. Thereby, the formative evaluation should focus on the utility, but also the reasons for non-use of the interventions [13].

Most face-to-face interventions used a participatory approach involving stakeholders such as nurses and physicians, who were indicated as potential recipients of the intervention, or who actively facilitate the implementation within the hospital. A possible reason for those strategies could be the delivery of interventions within the organization itself and the requirement of additional personnel resources for applying the interventions in person. These methods could streamline the implementation process and promote adoption of the intervention. However, Graham et al. [13] concluded that implementing digital health interventions differs from implementing face-to-face interventions. Therefore, a participatory approach is essential for both formats – one that involves not only nurses and physicians but also other stakeholders such as management, to ensure that intervention are developed or adapted to meet individuals needs and local conditions [13, 14, 53].

This raises the question of why stakeholder involvement is emphasized particularly in face-to-face interventions, while the focus in digital interventions tends to be on evaluation. In the case of digital interventions, the priority was formative evaluation or needs assessment rather than stakeholder involvement—particularly of nurses and physicians. This may be due to the fact that many digital interventions are already ‘evidence-informed.’ In contrast, face-to-face interventions often require broader stakeholder engagement for successful dissemination. Digital interventions, on the other hand, were typically disseminated by those already using them. For applying digital PTSD-related interventions, evaluative and iterative methods were most frequently analyzed. This iterative-evaluative approach not only enhanced the rapid delivery of psychological support for nurses and physicians but also allowed for scientific monitoring of the implementation process.

Furthermore, the implementation of a digital intervention required infrastructural changes prompted by the conditions of the pandemic. As evidence-based interventions were adapted to pandemic conditions and digital formats emerged, changes in how these interventions were delivered became inevitable. Witteveen et al. [50] concluded that the use of the digital format allowed a wider extension of services and thus more efficient adoption and utility. However, not only digital interventions but also some face-to-face interventions had to be adapted, for example, by modifying the location where it is provided and the used equipment [26, 42].

### Practical implications

Our results indicate that the applied methods to implement digital and face-to-face interventions for nurses and physicians differ.

Based on our research findings regarding the implementations of PTSD-related interventions for nurses and physicians working in a hospital, the following practical implications for decision-makers in hospitals can be derived:

1. Consider adopting and implementing a PTSD intervention in a digital format to improve compatibility and adaptability due to time and resource restrictions of nurses and physicians in a hospital.
2. Collect and submit ongoing feedback from the nurses and physicians regarding their experiences, acceptance and utilization of the PTSD intervention.
3. Deploy the feedback from nurses and physicians to enhance the adoption and sustainable use of the PTSD intervention.
4. Select and establish implementation facilitators who served as peers to coordinate the implementation of PTSD interventions on single wards or units within the hospital.
5. Offer ongoing educational sessions designed to train nurses and physicians in the application of the PTSD intervention.
6. Assure ongoing clinical supervision for all involved stakeholders in the implementation and intended use of the PTSD intervention.

These points may serve as recommendations for decision-makers to support the adoption and facilitate the implementation of interventions aimed at treating PTSD symptoms in hospital-based nurses and physicians.

## Limitations

This scoping review has several methodological and result-related strengths and limitations.

As the primary objective was to explore and map applied interventions that could be employed by nurses and physicians in the treatment of PTSD appeared during the unexpected COVID-pandemic, and to ascertain strategies for the implementation of these interventions, we did not conduct a critical appraisal. However, since the pandemic is over now, we strongly recommend the use of critical appraisals as well as assessment of risk of bias to investigate the quality of results in the future, for example as part of a systematic review.

Additionally, the review protocol was neither published nor registered with the Open Science Framework (OSF). To ensure transparency and replicability of the methodological steps during our scoping review, we followed the approach of Peters et al. [17] and the PRISMA-ScR guidelines.

Owing to time and other constraints, three databases were used for the data collection. Therefore, potentially relevant studies may have not been identified. However, with employing PubMed, PsychINFO, and CINAHL, we cover a broad search field of health, nursing, and implementation science, as well as psychology/psychiatry. Additionally, supplementary search options such as backward and forward citation screening and trial registry searches were applied to minimize the bias of the limited number of databases.

Further, we did not calculate the inter-rater reliability to ensure the quality of coding. Instead, one researcher performed the analysis in two iterations in exchanges with another researcher, and we present examples of coding in the paper to ensure a transparent and replicable procedure.

Regarding the limitation of results; these are based on a predefined population, concept and context, and may not reflect the general population. However, pre-defining the target population of PTSD interventions is important for the use of implementation strategies. This led to the focus on nurses and physicians as the primary group of professionals, and to analyze applied implementation strategies for each identified PTSD intervention. In contrast, because of the lack of evidence, studies that did not focus primarily on the specific population of nurses and physicians or the acute hospital setting were excluded. The implementation strategies did not differentiate between nurses and physicians, as the interventions identified were designed for interdisciplinary application.

Further, the results of our scoping review originate from articles published between 2020 and 2023. Most of the identified interventions were classified as evidence-informed as they were adapted to the COVID-19 pandemic conditions. Due to a lack of resources, we are not able to perform a literature update. Therefore, it might be that for most of the evidence-informed interventions, evidence of efficacy is now available. Besides, with our scoping review, we additionally intended to show in which way psychological support was provided for nurses and physicians working in hospitals in the period of the COVID-19 pandemic. The finding that most evidence-based interventions between 2020 and 2023 were adapted to digital formats in response to the pandemic—without being evaluated for effectiveness—is significant. It confirms that due to the increased mental health problems, the rapid delivery of psychological support was of priority.

## Conclusion

Our scoping review intended to identify interventions for PTSD symptoms in nurses and physicians in an acute hospital setting during the COVID-19 pandemic and the implementation strategies used to implement those interventions. The central research questions for this objective were as follows: What are the [1] interventions that address symptoms of post-traumatic stress disorder in hospital-based nurses and physicians during the COVID-19 pandemic? What are the [2] implementation strategies for the identified interventions?

Most PTSD-interventions during the COVID-19 pandemic between 2020 and 2023 have been adapted to existing conditions and developed as ‘evidence-informed’ interventions in a digital format to fit within the pandemic context with social distance and challenging working conditions. The effectiveness of these interventions are mainly not given due to the urgency and rapid development. Future research should address this research gap, and include subsequent systematic reviews with meta-analysis to strengthen the quality and effect of these interventions. This aspect is crucial for providing evidence-based guidance to hospital decision-makers for adopting and implementing PTSD-related interventions to prevent mental health issues in nurses and physicians—potentially as part of workplace health promotion programs.

Besides, we recommend considering the ethical aspects when assessing effectiveness studies, since nurses, physicians and other HCWs are vulnerable groups.

Given the urgent need to rapidly develop and implement PTSD-related interventions, researchers primarily employed implementation strategies that included evaluative components and actively involved stakeholders throughout the development and implementation process. Notably, none of the studies were conducted as implementation studies using implementation approaches or frameworks. Therefore the delivery of the intervention was described instead of the implementation itself. Further investigation should focus on the effectiveness of those strategies for the implementation of digital interventions, in contrast to face-to-face interventions due to implementation outcomes, like adoption, acceptability, and feasibility and sustainability.

## Supplementary Information

Additional File 1: Checklist of the PRISMA Extension for Scoping Reviews

Additional File 2: Research protocol

## Data Availability

No datasets were generated or analysed during the current study.

## Abbreviations

PTSD: Post-traumatic stress disorder
TIDieR: Template for intervention description and replication
ERIC: Framework Expert for Recommendations for Implementing Change
COVID-19: Coronavirus disease
HCW: Health care workers
CBT: Cognitive behavioral Therapy
DMHI: Digital Mental Health Interventions
JBI: Joanna Briggs Institute
ICD-10: International Statistical Classification of Diseases and Related Health Problems 10th Revision
PRESS: Peer Review of Electronic Search Strategies
APD: Anticipate-Plan-Deter
AIP: Adaptive Information Processing model
PTSD: Post-traumatic stress disorder

## References

[1] WHO. WHO Coronavirus (COVID-19) Dashboard World Health Organization 2021 https://covid19.who.int/. Accessed 16 Dec 2024.

[2] Lee BEC, Ling M, Boyd L, Olsson C, Sheen J. The prevalence of probable mental health disorders among hospital healthcare workers during COVID-19: a systematic review and meta-analysis. J Affect Disord. 2023. 10.1016/j.jad.2023.03.012. 36931567 10.1016/j.jad.2023.03.012 PMC10017178

[3] Ghahramani S, Kasraei H, Hayati R, Tabrizi R, Marzaleh MA. Health care workers’ mental health in the face of COVID-19: a systematic review and meta-analysis. Int J Psychiatry Clin Pract. 2022. 10.1080/13651501.2022.2101927. 35875844 10.1080/13651501.2022.2101927

[4] Duden GS, Reiter J, Paswerg A, Weibelzahl S. Mental health of healthcare professionals during the ongoing COVID-19 pandemic: a comparative investigation from the first and second pandemic years. BMJ Open. 2023. 10.1136/bmjopen-2022-067244. 36948559 10.1136/bmjopen-2022-067244 PMC10039975

[5] Andhavarapu S, Yardi I, Bzhilyanskaya V, Lurie T, Bhinder M, Patel P, et al. Post-traumatic stress in healthcare workers during the COVID-19 pandemic: A systematic review and meta-analysis. Psychiatry Res. 2022. 10.1016/j.psychres.2022.114890. 36260970 10.1016/j.psychres.2022.114890 PMC9573911

[6] WHO. International Statistical Classification of Diseases and Related Health Problems 10th Revision (ICD-10)-WHO Version for 2019-covid-expanded. Chapter V Mental and behavioral disorders (F00-F99) World Health Organization 2019 https://icd.who.int/browse10/2019/en#/F43.2. Accessed 16 Dec 2024.

[7] Evanoff BA, Strickland JR, Dale AM, Hayibor L, Page E, Duncan JG, et al. Work-related and personal factors associated with mental well-being during the COVID-19 response: survey of health care and other workers. J Med Internet Res. 2020. 10.2196/21366. 32763891 10.2196/21366 PMC7470175

[8] Chew NWS, Lee GKH, Tan BYQ, Jing M, Goh Y. A multinational, multicentre study on the psychological outcomes and associated physical symptoms among healthcare workers during COVID-19 outbreak. Brain, Behaviour, and Immunity 2020; 10.1016/j.bbi.2020.04.049. 10.1016/j.bbi.2020.04.049 PMC7172854 32330593

[9] Zace D, Hoxhaj I, Orfino A, Viteritti AM, Janiri L, Di Pietro ML. Interventions to address mental health issues in healthcare workers during infectious disease outbreaks: a systematic review. J Psychiatr Res. 2021. 10.1016/j.jpsychires.2021.02.019. 33636688 10.1016/j.jpsychires.2021.02.019 PMC7880838

[10] Wensing M, Sales A, Armstrong R, Wilson P. Implementation science in times of Covid-19. Implement Sci. 2020. 10.1186/s13012-020-01006-x. 32513292 10.1186/s13012-020-01006-x PMC7276954

[11] Proctor EK, Powell BJ, McMillen JC. Implementation strategies: recommendations for specifying and reporting Implementation Science. 2013. 10.1186/1748-5908-8-139. 24289295 10.1186/1748-5908-8-139 PMC3882890

[12] Powell BJ, Waltz TJ, Chinman MJ, Damschroder LJ, Smith JL, Matthieu MM, et al. A refined compilation of implementation strategies: results from the Expert Recommendations for Implementing Change (ERIC) project. Implement Sci. 2015. 10.1186/s13012-015-0209-1. 25889199 10.1186/s13012-015-0209-1 PMC4328074

[13] Graham AK, Lattie EG, Powell BJ, Lyon AR, Smith JD, Schueller SM, et al. Implementation strategies for digital mental health interventions in health care settings. Am Psychol. 2020. 10.1037/amp0000686. 33252946 10.1037/amp0000686 PMC7709140

[14] May CR, Johnson M, Finch T. Implementation, context and complexity. Implement Sci. 2016. 10.1186/s13012-016-0506-3. 27756414 10.1186/s13012-016-0506-3 PMC5069794

[15] Liyanage S, Addison S, Ham E, Hilton NZ. Workplace interventions to prevent or reduce post-traumatic stress disorder and symptoms among hospital nurses: a scoping review. J Clin Nurs. 2022. 10.1111/jocn.16076. 34636115 10.1111/jocn.16076

[16] Benavides-Gil G, Martínez-Zaragoza F, Fernández-Castro J, Sánchez-Pérez A, García-Sierra R. Mindfulness-based interventions for improving mental health of frontline healthcare professionals during the COVID-19 pandemic: a systematic review. Syst Rev. 2024. 10.1186/s13643-024-02574-5. 38902795 10.1186/s13643-024-02574-5 PMC11188518

[17] Peters MDJ, Marnie C, Tricco AC, Pollock D, Munn Z, Alexander L, et al. Updated methodological guidance for the conduct of scoping reviews. JBI Evid Synth. 2020. 10.11124/JBIES-20-00167. 33038124 10.11124/JBIES-20-00167

[18] Tricco AC, Lillie E, Zarin W, O'Brien KK, Colquhoun H, Levac D, et al. PRISMA Extension for Scoping Reviews (PRISMA-ScR): Checklist and Explanation. Ann Intern Med. 2018; 10.7326/M18-0850. 10.7326/M18-0850 30178033

[19] Nordhausen T. Hirt J RefHunter im neuen Webformat: Eine Plattform zur systematischen Literaturrecherche GMS Medizin. 2022. 10.3205/mbi000549.

[20] McGowan J, Sampson M, Salzwedel DM, Cogo E, Foerster V, Lefebvre C. PRESS Peer Review of Elektronic Search Strategies: 2015 Guideline Statement Journal of Clinical Epidemiology. 2016; 10.1186/s13643-017-0625-1. 10.1016/j.jclinepi.2016.01.021 27005575

[21] Cooper C, Booth A, Britten N, Garside R. A comparison of results of empirical studies of supplementary search techniques and recommendations in review methodology handbooks: a methodological review. Syst Rev. 2017. 10.1186/s13643-017-0625-1. 29179733 10.1186/s13643-017-0625-1 PMC5704629

[22] Ouzzani M, Hammady H, Fedorowicz Z, Elmagarmid A. Rayyan—a web and mobile app for systematic reviews. Syst Rev. 2016. 10.1186/s13643-016-0384-4. 27919275 10.1186/s13643-016-0384-4 PMC5139140

[23] Page MJ, McKenzie JE, Bossuyt PM, Boutron I, Hoffmann TC, Mulrow CD ea. The PRISMA 2020 statement: an updated guideline for reporting systematic reviews. BMJ. 2021; 10.1136/bmj.n71. 10.1136/bmj.n71 PMC8005924 33782057

[24] Hoffmann TC, Glasziou PR, Boutron I, Milne R, Perera R, Moher D, et al. Better reporting of interventions: the Template for Intervention Description and Replication (TIDieR) checklist and guide. BMJ. 2014. 10.1136/bmj.g1687. 24609605 10.1136/bmj.g1687

[25] Waltz TJ, Powell BJ, Matthieu MM, Damschroder LJ, Chinman MJ, Smith JL, et al. Use of concept mapping to characterize relationships among implementation strategies and assess their feasibility and importance: results from the expert recommendations for implementing change (ERIC) study. Implement Sci. 2015. 10.1186/s13012-015-0295-0. 26249843 10.1186/s13012-015-0295-0 PMC4527340

[26] Lefevre H, Stheneur C, Cardin C, Fourcade L, Fourmaux C, Tordjman E, et al. The Bulle: Support and Prevention of Psychological Decompensation of Health Care Workers During the Trauma of the COVID-19 Epidemic. J Pain Symptom Manage. 2021. 10.1016/j.jpainsymman.2020.09.023. 32961219 10.1016/j.jpainsymman.2020.09.023 PMC7836408

[27] Blake H, Bermingham F, Johnson G, Tabner A. Mitigating the psychological impact of COVID-19 on healthcare workers: a digital learning package. Int J Environ Res Public Health. 2020. 10.3390/ijerph17092997. 32357424 10.3390/ijerph17092997 PMC7246821

[28] Albott CS, Wozniak JR, McGlinch BP, Wall MH, Gold BS, Vinogradov S. Battle buddies: rapid deployment of a psychological resilience intervention for health care workers during the COVID-19 pandemic. Anesth Analg. 2020. 10.1213/ANE.0000000000004912. 32345861 10.1213/ANE.0000000000004912 PMC7199769

[29] Weiner L, Berna F, Nourry N, Severac F, Vidailhet P, Mengin AC. Efficacy of an online cognitive behavioral therapy program developed for healthcare workers during the COVID-19 pandemic: the REduction of STress (REST) study protocol for a randomized controlled trial. Trials. 2020. 10.1186/s13063-020-04772-7. 33087178 10.1186/s13063-020-04772-7 PMC7576984

[30] Dong L, Meredith LS, Farmer CM, Ahluwalia SC, Chen PG, Bouskill K, et al. Protecting the mental and physical well-being of frontline health care workers during COVID-19: study protocol of a cluster randomized controlled trial. Contemp Clin Trials. 2022. 10.1016/j.cct.2022.106768. 35470104 10.1016/j.cct.2022.106768 PMC9023359

[31] Sulaiman AH, Ahmad Sabki Z, Jaafa MJ, Francis B, Razali KA, Juares Rizal A, Mokhtar NH, Juhari JA, Zainal S, Ng CG. Development of a Remote Psychological First Aid Protocol for Healthcare Workers Following the COVID-19 Pandemic in a University Teaching Hospital, Malaysia. Healthcare (Basel). 2020 A.D.; 8. 10.3390/healthcare8030228. Cited in: PMID: 32722042. 10.3390/healthcare8030228 PMC7551586 32722042

[32] Wang Y, Duan Z, Peng K, Li D, Ou J, Wilson A, et al. Acute stress disorder among frontline health professionals during the COVID-19 outbreak: a structural equation modeling investigation. Psychosom Med. 2021. 10.1097/psy.0000000000000851. 32815855 10.1097/PSY.0000000000000851

[33] Morina N, Weilenmann S, Dawson KS, Ernst J, Zanitti Z, von Känel R, et al. RECHARGE - A Brief Psychological Intervention to Build Resilience in Health Care Workers During the COVID-19 Pandemic: Study Protocol for a Randomized Controlled Trial Preprint 2021; 10.21203/rs.3.rs-212942/v1.

[34] Trottier K, Monson CM, Kaysen D, Wagner AC, Pun C, Abbey SE. Development of RESTORE: an online intervention to improve mental health symptoms associated with COVID-19-related traumatic and extreme stressors. Eur J Psychotraumatol. 2021. 10.1080/20008198.2021.1984049. 34745446 10.1080/20008198.2021.1984049 PMC8567930

[35] Sagaltici E, Saydam RB, Cetinkaya M, Şahin ŞK, Küçük SH, Müslümanoğlu AY. Burnout and psychological symptoms in healthcare workers during the COVID-19 pandemic: comparisons of different medical professions in a regional hospital in Turkey. Work (Reading, Mass). 2022. 10.3233/WOR-210517. 35634831 10.3233/WOR-210517

[36] Kanellopoulos D, Solomonov N, Ritholtz S, Wilkins V, Goldman R, Schier M, et al. The Copenyp program: a model for brief treatment of psychological distress among healthcare workers and hospital staff. Gen Hosp Psychiatry. 2021. 10.1016/j.genhosppsych.2021.09.002. 34536798 10.1016/j.genhosppsych.2021.09.002 PMC8420093

[37] Solomonov N, Kanellopoulos D, Grosenick L, Wilkins V, Goldman R, Ritholtz S, et al. Copenyp: a brief remote psychological intervention reduces health care workers’ depression and anxiety symptoms during COVID-19 pandemic. World Psychiatry. 2022. 10.1002/wps.20946. 35015344 10.1002/wps.20946 PMC8751568

[38] Trottier K, Monson CM, Kaysen D, Wagner AC, Liebman RE, Abbey SE. Initial findings on RESTORE for healthcare workers: an internet-delivered intervention for COVID-19-related mental health symptoms. Transl Psychiatry. 2022. 10.1038/s41398-022-01965-3. 35650179 10.1038/s41398-022-01965-3 PMC9157042

[39] Fogliato E, Invernizzi R, Maslovaric G, Fernandez I, Rigamonti V, Lora A, et al. Promoting mental health in healthcare workers in hospitals through psychological group support with eye movement desensitization and reprocessing during COVID-19 pandemic: an observational study. Front Psychol. 2022. 10.3389/fpsyg.2021.794178. 35153919 10.3389/fpsyg.2021.794178 PMC8829464

[40] Bureau R, Bemmouna D, Faria CGF, Goethals A-AC, Douhet F, Mengin AC, et al. My health too: Investigating the feasibility and the acceptability of an internet-based cognitive-behavioral therapy program developed for healthcare workers. Front Psychol. 2021. 10.3389/fpsyg.2021.760678. 34925163 10.3389/fpsyg.2021.760678 PMC8677821

[41] Wang L, Norman I, Xiao T, Li Y, Li X, Leamy M. Evaluating a psychological first aid training intervention (preparing me) to support the mental health and wellbeing of Chinese healthcare workers during healthcare emergencies: protocol for a randomized controlled feasibility trial. Front Psychiatry. 2022. 10.3389/fpsyt.2021.809679. 35153867 10.3389/fpsyt.2021.809679 PMC8830777

[42] Hannig C, Lotzin A, Milin S, Schäfer I. Stress- und Traumaprävention für Beschäftigte im Gesundheitsbereich = Stress and trauma prevention for employees in the health sector. Trauma Gewalt. 2021. 10.21706/tg-15-3-232.

[43] Mellins CA, Mayer LES, Glasofer DR, Devlin MJ, Albano AM, Nash SS, et al. Supporting the well-being of health care providers during the COVID-19 pandemic: The copecolumbia response. Gen Hosp Psychiatry. 2020. 10.1016/j.genhosppsych.2020.08.013. 33059217 10.1016/j.genhosppsych.2020.08.013 PMC7480793

[44] Serrano-Ripoll MJ, Meneses-Echavez JF, Ricci-Cabello I, Fraile-Navarro D, Fiol-deRoque MA, Pastor-Moreno G, et al. Impact of viral epidemic outbreaks on mental health of healthcare workers: a rapid systematic review and meta-analysis. J Affect Disord. 2020. 10.1016/j.jad.2020.08.034. 32861835 10.1016/j.jad.2020.08.034 PMC7443314

[45] Dumarkaite A, Truskauskaite I, Andersson G, Jovarauskaite L, Jovaisiene I, Nomeikaite A, et al. The efficacy of the internet-based stress recovery intervention FOREST for nurses amid the COVID-19 pandemic: a randomized controlled trial. Int J Nurs Stud. 2023. 10.1016/j.ijnurstu.2022.104408. 36527859 10.1016/j.ijnurstu.2022.104408 PMC9684088

[46] Jovarauskaite L, Dumarkaite A, Truskauskaite-Kuneviciene I, Jovaisiene I, Andersson G, Kazlauskas E. Internet-based stress recovery intervention FOREST for healthcare staff amid COVID-19 pandemic: study protocol for a randomized controlled trial. Trials. 2021. 10.1186/s13063-021-05512-1. 34419114 10.1186/s13063-021-05512-1 PMC8380103

[47] Fiol-DeRoque MA, Serrano-Ripoll MJ, Jiménez R, Zamanillo-Campos R, Yáñez-Juan AM, Bennasar-Veny M, Leiva A, Gervilla E, García-Buades ME, García-Toro M, et al. A Mobile Phone-Based Intervention to Reduce Mental Health Problems in Health Care Workers During the COVID-19 Pandemic (PsyCovidApp): Randomized Controlled Trial. JMIR Mhealth Uhealth. 2021 A.D.; 9:e27039. 10.2196/27039. Cited in: PMID: 33909587. 10.2196/27039 PMC8133164 33909587

[48] Proctor E, Silmere H, Raghavan R, Hovmand P, Aarons G, Bunger A, et al. Outcomes for implementation research: conceptual distinctions, measurement challenges, and research agenda. Adm Policy Ment Health. 2011. 10.1007/s10488-010-0319-7. 20957426 10.1007/s10488-010-0319-7 PMC3068522

[49] Pollock A, Campbell P, Cheyne J, Cowie J, Davis B, McCallum J, et al. Interventions to support the resilience and mental health of frontline health and social care professionals during and after a disease outbreak, epidemic or pandemic: a mixed methods systematic review. Cochrane Database Syst Rev. 2020. 10.1002/14651858.CD013779. 33150970 10.1002/14651858.CD013779 PMC8226433

[50] Witteveen AB, Young S, Cuijpers P, Ayuso-Mateos JL, Barbui C, Bertolini F, et al. Remote mental health care interventions during the COVID-19 pandemic: an umbrella review. Behav Res Ther. 2022. 10.1016/j.brat.2022.104226. 36410111 10.1016/j.brat.2022.104226 PMC9661449

[51] Hao Q, Wang D, Xie M, Tang Y, Dou Y, Zhu L, et al. Prevalence and risk factors of mental health problems among healthcare workers during the COVID-19 pandemic: a systematic review and meta-analysis. Front Psychiatry. 2021. 10.3389/fpsyt.2021.567381. 34211406 10.3389/fpsyt.2021.567381 PMC8239157

[52] Robins-Browne K, Lewis M, Burchill LJ, Gilbert C, Johnson C, O’Donnell M, et al. Interventions to support the mental health and well-being of front-line healthcare workers in hospitals during pandemics: an evidence review and synthesis. BMJ Open. 2022. 10.1136/bmjopen-2022-061317. 36344001 10.1136/bmjopen-2022-061317 PMC9644079

[53] Greenhalgh T, Robert G, Macfarlane F, Bate P, Kyriakidou O. Diffusion of innovations in service organizations: systematic review and recommendations. Milbank Q. 2004. 10.1111/j.0887-378x.2004.00325.x. 15595944 10.1111/j.0887-378X.2004.00325.x PMC2690184

