# Supplementary material for: Implementing PTSD interventions for hospital nurses and physicians during COVID-19: A scoping review
Source: Arch Public Health. 2025 Oct 2;83:235. doi: 10.1186/s13690-025-01706-0 (PMC12492515; doi:10.1186/s13690-025-01706-0)
Supplement: Supplementary file 2 — Additional File 2: Research protocol [file 13690_2025_1706_MOESM2_ESM.pdf]

Additional File 2: Research protocol

Research protocol

Interventions treating symptoms of post-traumatic stress disorder among hospital-based nurses and physicians during the COVID-19 pandemic

Content

List of Tables 3
1. Research question 4
2. Inclusion and exclusion criteria 4
3. Search strategies 5
4. Identification of synonym search terms 6
5. Identification of keywords 7
6. Search string development 9
7. Review search strings 17
8. Conducting and documentation of the search 21
9. Supplementary search options 31
10. Title-Abstract- and full-text-screening 40
11. References 41
12. Supplement I Flowchart 43
13 Supplement II Bibliographic information of the articles included for data extraction 44

List of Tables

Table 1: Inclusion and exclusion criteria (own visualization) 4
Table 2: Synonym search terms (own presentation based on Nordhausen and Hirt (7)) . 6
Table 3: Identified key words (own representation based on Nordhausen and Hirt (7)) . 7
Table 4: Search string for the database MEDLINE via PubMed (own representation based on Nordhausen and Hirt (7)) 9
Table 5: Search filters 12
Table 6: Rehearsal with the generated search string performed in MEDLINE via PubMed based 12
Table 7: Search string syntax for MEDLINE via PubMed and Psych INFO via EBSCO (own representation) 14
Table 8: Search filters 21
Table 9: Documentation of the search (own representation based on Nordhausen and Hirt (7)) 22
Table 10: Documentation of the search in PsychINFO via EBSCO (own representation) 26
Table 11: Presentation of research findings from the supplementary search in study register (own visualization). 31
Table 12: Presentation of results from the backward citation tracking (own visualization). 33
Table 13: Results from a 'hand search' in Google scholar based on resulted psychological interventions from a systematic review by Hooper et al. (11) (own visualization). 33
Table 14: Documentation of the search in CINAHL via EBSCO (own representation) 35

1. Research question

The aim of this systematic literature research is to explore interventions treating symptoms of post-traumatic stress disorder (PTSD) among nurses and physicians working in an acute hospital setting during the COVID-19 pandemic. Further, to identify strategies to implement these interventions.

The central research question guiding this systematic literature search is:

What are the [1] interventions that address symptoms of post-traumatic stress disorder in hospital-based nurses and physicians during the COVID-19 pandemic? What are the [2] implementation strategies for the identified interventions?

2. Inclusion and exclusion criteria

Based on the **PCC**-elements (**P**opulation, **C**oncept, **C**ontext) (1) the in- and exclusion criteria are defined in Table 1 below:

Table 1: Inclusion and exclusion criteria (own visualization)

Criteria	**Definition (inclusion)**	**Definition (exclusion)**
Population	Nurses and physicians with symptoms of post-traumatic stress disorder (PTSD) as target population for the evidence-based interventions	Other professions (e.g., Community Health Nurses, physiotherapist, respiratory therapist)
Concept	Interventions related to PTSD, specifically one of these symptoms showing in nurses and physicians working at a hospital[1]:<br>▪ **Intrusion** (e.g., intrusive thoughts, involuntary memories, distressing dreams, flashbacks)<br>▪ **Avoidance** (e.g., avoiding people, places, activities, objects and situations)<br>▪ **Alterations in cognition and mood** (e.g., feeling detached or estranged from others, unable to experience positive emotions)<br>▪ **Alterations in arousal and reactivity** (e.g., insomnia)<br>▪ Distress (psychological)<br>▪ Anhedonia<br>▪ Anxiety<br>▪ Depression<br>▪ Suicidal ideations<br>▪ Acute stress (disorder)<br>Strategies used to implement these interventions	Non-PTSD related interventions

Context	Acute somatic hospital setting during the COVID-19 pandemic period	Specialized clinics such as mental/ psychiatric hospital
Types of evidence sources	Evaluation and implementation studies, study protocol, feasibility studies, concept articles	Reviews
Other	Languages: German and English<br>Year: 2020- 2023	Published before 2020 and after 2023

[1] (Taylor-Desir, 2022; WHO, 2019)

<u>Rationale for in- and exclusion criteria:</u>

Population

Physicians and nurses who were working/ work during the COVID-19 pandemic in period from 2019-2023. Couarraze et al. (2) stated in an international survey that the levels of stress from healthcare professionals was 25.8% higher during the pandemic than the general population.

Concept

Saragih et al. (3) ascertain that during the pandemic globally the prevalence of PTSD in physicians and nurses is as a result from a pooled analysis 49%. The researchers included 38 studies in the year 2020, which provided a comprehensive overview of the effects of the pandemic, globally. Across all studies, a total of 53,784 participants were included. Of it, 27,9% were doctors and 43,7% nurses.

Context

Context of interest is the COVID-19 pandemic, because this and any kind of disease outbreak could increase risk of mental health problems among healthcare workers in short or long term (4). Only acute somatic hospital-based physicians and nurses are the focus of this study, because they have been caring to a larger extent for patients infected with COVID-19. This kind of environment is associated with a higher prevalence of stress, anxiety, and burnout among staff (5).

3. Search strategies

Based on the PCC-elements, MEDLINE (via PubMed) is chosen because of the wide range of topics in the field of health and the use of research methods. In addition, PsychINFO (via EBSCO) is specialized in psychiatric/ mental health issues, which is necessary for getting all relevant literature related to the objective.

An initial limited search will be conducted in the two databases to identify synonym search terms and key words for each. Second, search strings for each database will be developed to conduct systematic literature research. Third, the search string for MEDLINE (via PubMed), will be verify using the Peer Review of Electronic Search Strategies (PRESS) (6). Fourth, search string for PsychINFO (via EBSCO) will be developed. Finally, further search possibilities as a supplemental limited search in CINAHL (via EBSCO), citation tracking will be conducted (1).

4. Identification of synonym search terms

Table 2: Synonym search terms (own presentation based on Nordhausen and Hirt (7))

**Criteria/ search component**	**Search terms**
**Population**	Physician/Physicians<br>Medical Doctor<br>Nurse/Nurses<br>Nursing staff<br>Medical staff<br>Professional Caregivers<br>Registered nurse<br>Nurse practitioner<br>Advanced practice nurse<br>health care workers<br>health workers<br>health-care workers<br>health care professionals<br>frontline healthcare workers
**Concept**	intervention<br><br>post-traumatic stress disorder<br>PTSD<br>Acute stress disorder<br>Psychological Distress
**Context**	hospital<br>acute hospital<br>clinic<br>acute setting<br>hospital setting<br><br>COVID-19<br>COVID-19 pandemic

<u>Rationale:</u>

Concept:

Liang et al. (8) shows that psychological distress could lead to symptoms of PTSD.

5. Identification of keywords

Table 3: Identified key words (own representation based on Nordhausen and Hirt (7))

Search terms	Search component	keyword (MEDLINE via PubMed)	keyword (Psych INFO via EBSCO)
Physician<br>Physicians<br>Doctor<br>Physician Assistant<br>Nurse<br>Nurses<br>Nursing staff<br>Caregivers<br>Registered nurse<br>Nurse practitioner<br>Advanced practice nurse<br>health care workers<br>health workers<br>health-care workers<br>health care professionals<br>frontline healthcare workers	**Population**	Nurse Clinicians<br>Nurse Specialists<br>Physician Assistants<br>Nurse Specialists<br>Nursing Staff, Hospital<br>Health Personnel	Physicians<br>Medical Personnel<br>Clinicians<br>Nurses<br>Caregivers<br>Professional Personnel<br>Medical Personnel<br>Frontline Employees<br>Health Personnel
intervention<br>post-traumatic stress disorder<br>PTSD<br>Acute stress disorder<br>Psychological Distress	**Concept**	Stress Disorders, Post-Traumatic<br>Psychological Distress<br>Stress Disorders, Traumatic, Acute	Posttraumatic Stress Disorder<br>Posttraumatic Stress<br>Stress and Trauma related disorders<br>Acute Stress Disorder<br>Acute Stress<br>Caregiver burden
hospital<br>hospitals<br>acute hospital<br>clinic<br>acute setting	**Context**	Hospitals<br>Subacute care<br>COVID-19	Hospitals<br>Clinics<br>Treatment Facilities

**Search terms**	**Search component**	**keyword (MEDLINE via PubMed)**	**keyword (Psych INFO via EBSCO)**
hospital setting<br>COVID-19<br>COVID-19 pandemic			

Rationale for keywords in MEDLINE via PubMed

Context:

Subacute care is defined as: *“Medical and skilled nursing services provided to patients who are not in an acute phase of an illness but who require a level of care higher than that provided in a long-term care setting. (JCAHO, Lexikon, 1994)”*. Based on that, in relation to the objective this keyword is included.

6. Search string development

According to the recommendation of Nordhausen and Hirt (7) first the search string for the database MEDLINE via PubMED will be developed. In the subsequent step, this will be tested in a trial run following the recommendation so that an adjustment can be made and thus the effort for the PsychINFO via EBSCO database is minimized.

Table 4: Search string for the database MEDLINE via PubMed (own representation based on Nordhausen and Hirt (7))

Search components	Searchstring MEDLINE via PubMed	Searchstring PsychINFO via EBSCO
**Population**	Physician* [TIAB]<br>OR<br>Doctor [TIAB]<br>OR<br>“Physician Assistant” [TIAB]<br>OR<br>Nurs* [TIAB]<br>OR<br>“Nursing staff” [TIAB]<br>OR<br>Caregiver* [TIAB]<br>OR<br>“Registered nurse” [TIAB]<br>OR<br>“Nurse practitioner” [TIAB]<br>OR<br>“Advanced practice nurse” [TIAB]<br>OR<br>“health care workers” [TIAB]<br>OR<br>“health workers” [TIAB]<br>OR<br>“health-care workers” [TIAB]<br>OR<br>“health care professionals” [TIAB]	

Search components	Searchstring MEDLINE via PubMed	Searchstring PsychINFO via EBSCO
	OR<br>“frontline healthcare workers” [TIAB]<br>OR<br>“Nurse Clinicians” [MH]<br>OR<br>“Nurse Specialists” [MH]<br>OR<br>“Physician Assistants” [MH]<br>OR<br>“Nurse Specialists” [MH]<br>OR<br>“Nursing Staff, Hospital” [MH]<br>OR<br>“Health Personnel” [MH]	
	**AND**	**AND**
**Search component 2**	Intervention* [TIAB]	
	**AND**	**AND**
	“post-traumatic stress disorder” [TIAB]<br>OR<br>PTSD [TIAB]<br>OR<br>“Acute Stress Disorder” [TIAB]<br>OR<br>“Stress Disorders, Post-Traumatic” [MH]<br>OR<br>“Psychological Distress” [MH]<br>OR<br>“Stress Disorders, Traumatic, Acute” [MH]	
	**AND**	**AND**
**Search component 3**	Hospital* [TIAB]<br>OR	

Search components	Searchstring MEDLINE via PubMed	Searchstring PsychINFO via EBSCO
	"acute hospital" [TIAB]<br>OR<br>clinic* [TIAB]<br>OR<br>"acute setting" [TIAB]<br>OR<br>"hospital setting" [TIAB]<br>OR<br>Hospitals [MH]<br>OR<br>"subacute care" [MH]	
	**AND**	**AND**
	COVID-19 [TIAB]<br>OR<br>"COVID-19 pandemic" [TIAB]<br>OR<br>COVID-19 [MH]	

The rehearsal with the generated search string was performed on 14th April 2023 in MEDLINE via PubMed (the search string was stored online).

The following search filters were used:

Table 5: Search filters

**Search filters:**	
**Publication Date**	2020-2023
**Language**	German, English
**Spezies**	Humans

Table 6: Rehearsal with the generated search string performed in MEDLINE via PubMed based

**Search number**	**Input**	**Number of hits**
1	Physician* [TIAB]	455,043
2	Doctor [TIAB]	66,984
3	"Physician Assistant" [TIAB]	1,953
4	Nurs* [TIAB]	527,820
5	"Nursing staff" [TIAB]	14,536
6	Caregiver* [TIAB]	89,622
7	"Registered nurse" [TIAB]	4,290
8	"Nurse practitioner" [TIAB]	6,987
9	"Advanced practice nurse" [TIAB]	1,124
10	"health care workers" [TIAB]	17,148
11	"health workers" [TIAB]	21,641
12	"health-care workers" [TIAB]	17,148
13	"health care professionals" [TIAB]	28,117
14	“frontline healthcare workers” [TIAB]	506
15	"Nurse Clinicians" [MH]	8,506
16	"Nurse Specialists" [MH]	19,488
17	"Physician Assistants" [MH]	6,403
18	"Nursing Staff, Hospital" [MH]	47,860
19	"Health Personnel" [MH]	606,399
20	#1 OR #2 OR #3 OR #4 OR #5 OR #6 OR #7 OR #8 OR #9 OR #10 OR #11 OR #12 OR #13 OR #14 OR #15 OR #16 OR #17 OR #18 OR #19	1,448,565

Search number	Input	Number of hits
21	Intervention* [TIAB]	1,303,362
22	"post-traumatic stress disorder" [TIAB]	15,956
23	PTSD [TIAB]	32,920
24	"Acute Stress Disorder" [TIAB]	779
25	"Psychological Distress" [TIAB]	27,502
26	"Stress Disorders, Post-Traumatic" [MH]	40,679
27	"Psychological Distress" [MH]	6,603
28	"Stress Disorders, Traumatic, Acute" [MH]	536
29	#22 OR #24 OR #25 OR #26 OR #27 OR #28	83,870
30	#21 AND #29	17,259
31	Hospital* [TIAB]	1,605,107
32	"acute hospital" [TIAB]	4,303
33	clinic* [TIAB]	5,160,437
34	"acute setting" [TIAB]	2,693
35	"hospital setting" [TIAB]	14,161
36	Hospitals [MH]	314,785
37	"Subacute care" [MH]	1,411
38	#31 OR #32 OR #33 OR #34 OR #35 OR #36 OR #37	6,264,657
39	COVID-19 [TIAB]	307,395
40	"COVID-19 pandemic" [TIAB]	115,894
41	COVID-19 [MH]	217,887
42	#39 OR #40 OR #41	335,265
43	#20 AND #30 OR #38 OR #42	**192**
Filter	*Humans, English, German*	**131**

Adjustment of the search string syntax

Table 7: Search string syntax for MEDLINE via PubMed and Psych INFO via EBSCO (own representation)

Search component	Search string MEDLINE via PubMed	Search string PsychINFO via EBSCO
**Population**	Physician* [TIAB]<br>OR<br>Doctor [TIAB]<br>OR<br>“Doctor of medicine” [TIAB]<br>OR<br>“Physician Assistant” [TIAB]<br>OR<br>Nurs* [TIAB]<br>OR<br>“Nursing staff” [TIAB]<br>OR<br>Caregiver* [TIAB]<br>OR<br>“Registered nurse” [TIAB]<br>OR<br>“Nurse practitioner” [TIAB]<br>OR<br>“Advanced practice nurse” [TIAB]<br>OR<br>“health care workers” [TIAB]<br>OR<br>“health workers” [TIAB]<br>OR<br>“health-care workers” [TIAB]<br>OR<br>“health care professionals” [TIAB]<br>OR<br>“frontline healthcare workers” [TIAB]<br>OR	(TI Physician* OR AB Physician* OR TI Doctor OR AB Doctor OR TI “Doctor of medicine” OR AB “Doctor of medicine” OR TI “Physician Assistant” OR AB “Physician Assistant” OR TI Nurs* OR AB Nurs* OR TI “Nursing staff” OR AB “Nursing staff” OR TI Caregiver* OR AB Caregiver* OR TI “Registered nurse” OR AB “Registered nurse” OR TI “Nurse practitioner” OR AB “Nurse practitioner” OR TI “Advanced practice nurse” OR AB “Advanced practice nurse” OR TI “health care workers” OR AB “health care workers” OR TI “health workers” OR AB “health workers” OR TI “health-care workers” OR AB “health-care workers” OR TI “health care professionals” OR AB “health care professionals” OR TI “frontline healthcare workers” OR AB “frontline healthcare workers” OR DE Health Personnel OR DE Physicians OR DE Medical Personnel OR DE Clinicians OR DE Nurses OR DE Caregivers OR DE Professional Personnel OR DE Medical Personnel OR DE Frontline Employees)

Search component	Search string MEDLINE via PubMed	Search string PsychINFO via EBSCO
	Physicians [MH]<br>OR<br>medical staff, hospital [MH]<br>OR<br>Nurses [MH]<br>OR<br>Nurse Practitioner [MH]<br>OR<br>Nursing Staff [MH]<br>OR<br>Caregivers [MH]<br>OR<br>Nurse Clinicians [MH]<br>OR<br>Nurse Specialists [MH]<br>OR<br>Physician Assistants [MH]<br>OR<br>Nurse Specialists [MH]<br>OR<br>Nursing Staff, Hospital [MH]<br>OR<br>Health Personnel [MH]	
	**AND**	**AND**
**Concept**	Intervention* [TIAB]	(TI intervention* OR AB intervention* OR DE intervention)
	**AND**	**AND**
	“post-traumatic stress disorder” [TIAB]<br>OR<br>PTSD [TIAB]<br>OR<br>“Acute Stress Disorder” [TIAB]<br>OR	(TI “post-traumatic stress disorder” OR AB “post-traumatic stress disorder” OR TI PTSD OR AB PTSD OR TI “Acute Stress Disorder” OR AB “Acute Stress Disorder” OR TI “Psychological Distress” OR AB “Psychological Distress” OR DE “Posttraumatic Stress Disorder” OR DE “Posttraumatic Stress” OR DE Trauma OR DE “Acute Stress

Search component	Search string MEDLINE via PubMed	Search string PsychINFO via EBSCO
	"Psychological Distress" [TIAB]<br>OR<br>"Stress Disorders, Post-Traumatic" [MH]<br>OR<br>"Psychological Distress" [MH]<br>OR<br>"Stress Disorders, Traumatic, Acute" [MH]	Disorder" OR DE "Stress and Trauma related disorders" OR DE "Caregiver burden")
**Context**	Hospital* [TIAB]<br>OR<br>"acute hospital" [TIAB]<br>OR<br>clinic* [TIAB]<br>OR<br>"acute setting" [TIAB]<br>OR<br>"hospital setting" [TIAB]<br>OR<br>Hospitals [MH]	(TI Hospital* OR AB Hospital* OR TI "acute hospital" OR AB "acute hospital" OR TI clinic* OR AB clinic* OR TI "acute setting" OR AB "acute setting" OR TI "hospital setting" OR AB "hospital setting" OR DE Hospitals OR DE Clinics OR DE Treatment Facilities)
	**AND**	**AND**
	COVID-19 [TIAB]<br>OR<br>"COVID-19 pandemic" [TIAB]<br>OR<br>COVID-19 [MH]	(TI COVID-19 OR AB COVID-19 OR TI "COVID-19 pandemic" OR AB "COVID-19 pandemic" OR DE COVID-19)

7. Review search strings

The creation and review of the search strings for MEDLINE via PubMed and PsychINFO via EBSCO, is done using the PRESS criteria (6). This tool is recommended by Nordhausen and Hirt (7) for reviewing search strings. The authors describe that not having an external person doing the review resulted in a less fineness. However, they also emphasize that with a high degree of (self-) reflection and conscientiousness, this consequence can be minimized to a large extent (7). In addition, the syntax was discussed with the supervisors.

At some points rationales are given to make the decisions behind the answers transparent and to show the reflective approach.

Search string validate
Name: Prof. Dr. phil. Martina Roes
Datum: 14th April 2023

<u>Translation research question(s)</u>

Does the search strategy fit my research question?

☒ Yes
☐ No

Are the search components clearly defined and delimited from each other?

☒ Yes
☐ No

Are there too few or too many search components?

☐ Yes
☒ No

Rationale:
In component **C**(onsept) it´s important to have to different components to the objective, respectively. Same in component **C**(ontext).

Are the search components too specific or too sensitive?

☐ Yes
☒ No

Are there too few or too many search hits?

☐ Yes
☒ No

Rationale:

With regard to the objective and the number of databases, 92 hits are generated in PubMed (via MEDLINE). The components are specific, but due to the research question.

<u>Operators</u>

Are the operators used correctly?

☒ Yes
☐ No
☐ Not applicable

Are the brackets in the search string placed correctly?

☐ Yes
☒ No
☐ Not applicable

Rationale:
The particulary search components were not be in brackets ( ).

Is it likely that operator NOT will lead to exclusion of potentially relevant publications?

☐ Yes
☐ No
☒ Not applicable

Rationale:
It was not necessary to use the operator NOT.

Could the use of word spacing operators lead to a more precise search result?

☐ Yes
☐ No
☒ Not applicable

Rationale:
Word spacing operators cannot be used in MEDLINE via PubMed and in PsychINFO via EBSCO lead the use of spacing operators to slightly number of hits.

Were word spacing operators used with an appropriate word spacing number?

☐ Yes
☐ No
☒ Not applicable

<u>Keywords (MeSH-terms/ Index terms)</u>

Are all keywords relevant?

☐ Yes
☒ No
☐ Not applicable

Are relevant keywords missing?

☐ Yes
☒ No
☐ Not applicable

Are subordinate or superordinate keywords too broad or too close?

☐ Yes
☒ No
☐ Not applicable

Were keywords properly expanded to include underlying keywords?

☒ Yes
☐ No
☐ Not applicable

Rationale:
In MEDLINE via PubMed with the "search command" [MH] all subheadings are included as well as underlying keywords. In PsychINFO via EBSCO every single keyword has to list separate with the "search command" [DE].

Were the subheadings correctly assigned to the keywords?

☒ Yes
☐ No
☐ Not applicable

Have keywords and catchwords been defined for each search component?

☒ Yes
☐ No
☐ Not applicable

<u>Keywords</u>

Do all keywords contain all **relevant** word combinations?

☒ Yes
☐ No
☐ Not applicable

Are all synonyms defined?

☒ Yes
☐ No
☐ Not applicable

Are placeholders inserted correctly?

☐ Yes
☒ No
☐ Not applicable

Is the truncation/ "Wildcard" placed too early or too late?

☐ Yes
☒ No
☐ Not applicable

Are acronyms and abbreviations used appropriately and are the spelled-out terms also integrated?

☒ Yes
☐ No
☐ Not applicable

Are the keywords too specific or too sensitive?

☐ Yes
☒ No
☐ Not applicable

Are the "search commands" set correctly?

☒ Yes
☐ No
☐ Not applicable

<u>Notation, syntax and number of lines</u>

Are there any spelling mistakes?

☐ Yes
☒ No

Are there any errors in the syntax?

☒ Yes
☐ No

Rationale:
Between two keywords the operator [OR] was missing.

Have all search components been integrated into the search string?

☒ Yes
☐ No

<u>Limitations and search filters</u>

Were limits and/ or search filters used appropriately and correctly?

☒ Yes
☐ No
☐ Not applicable

Are the limitations and/ or search filers indexed in the respective database?

☒ Yes
☐ No
☐ Not applicable

Are limits and/ or search filters missing?

- ☐ Yes
- ☒ No
- ☐ Not applicable

Rationale:
The criteria “source” leaves open, because of low number of resulted hits in the two databases.

8. Conducting and documentation of the search

The search filters listed in the following table are applied for the search in the subject databases MEDLINE via PubMed and CINAHL, partly derived from the inclusion and exclusion criteria:

Table 8: Search filters

**Search filter**	
**Publication Date**	2019-2023
**Language**	English, German, dutch
**Spezies**	Human

Search in MEDLINE via PubMed conducted at 14th April 2023 (search string is record online)

Table 9: Documentation of the search (own representation based on Nordhausen and Hirt (7))

Search component	Search number	Feed	Number of hits	Search string for MEDLINE via PubMed
**Population**	1	Physician* [TIAB]	455,043	(((((((((((((((((Physician* [TIAB]) OR (Doctor [TIAB])) OR ("Physician Assistant" [TIAB])) OR (Nurs* [TIAB])) OR ("Nursing staff" [TIAB])) OR (Caregiver* [TIAB])) OR ("Registered nurse" [TIAB])) OR ("Nurse practitioner" [TIAB])) OR ("Advanced practice nurse" [TIAB])) OR ("health care workers" [TIAB])) OR ("health workers" [TIAB])) OR ("health-care workers" [TIAB])) OR ("health care professionals" [TIAB])) OR ("frontline healthcare workers" [TIAB])) OR ("Nurse Clinicians" [MH])) OR ("Physician Assistants" [MH])) OR ("Nursing Staff, Hospital" [MH])) OR ("Health Personnel" [MH])) OR ("Nurse Specialists" [MH])
	2	Doctor [TIAB]	66,984	
	3	"Physician Assistant" [TIAB]	1,953	
	4	Nurs* [TIAB]	527,820	
	5	"Nursing staff" [TIAB]	14,536	
	6	Caregiver* [TIAB]	89,622	
	7	"Registered nurse" [TIAB]	4,290	
	8	"Nurse practitioner" [TIAB]	6,987	
	9	"Advanced practice nurse" [TIAB]	1,124	
	10	"health care workers" [TIAB]	17,148	
	11	"health workers" [TIAB]	21,641	
	12	"health-care workers" [TIAB]	17,148	
	13	"health care professionals" [TIAB]	28,117	
	14	"frontline healthcare workers" [TIAB]	506	
	15	"Nurse Clinicians" [MH]	8,506	
	16	"Physician Assistants" [MH]	6,403	
	17	"Nursing Staff, Hospital" [MH]	47,860	
	18	"Health Personnel" [MH]	606,399	
	19	"Nurse Specialists" [MH]	19,488	
	20	#1 OR #2 OR #3 OR #4 OR #5 OR #6 OR #7 OR #8 OR #9 OR #10 OR #11 OR #12 OR #13 OR #14 OR #15 OR #16 OR #17 OR #18 OR #19	1,448,565	

Search component	Search number	Feed	Number of hits	Search string for MEDLINE via PubMed
	21	Intervention* [TIAB]	1,303,632	
Concept	22	"post-traumatic stress disorder" [TIAB]	15,956	(((((("post-traumatic stress disorder" [TIAB]) OR (PTSD [TIAB])) OR ("Acute Stress Disorder" [TIAB])) OR ("Psychological Distress" [TIAB])) OR ("Stress Disorders, Post-Traumatic" [MH])) OR ("Psychological Distress" [MH])) OR ("Stress Disorders, Traumatic, Acute" [MH])
	23	PTSD [TIAB]	32,920	
	24	"Acute Stress Disorder" [TIAB]	779	
	25	"Psychological Distress" [TIAB]	27,502	
	26	"Stress Disorders, Post-Traumatic" [MH]	40,679	
	27	"Psychological Distress" [MH]	6,603	
	28	"Stress Disorders, Traumatic, Acute" [MH]	536	
	29	#22 OR #23 OR #24 OR #25 OR #26 OR #27 OR #28	83,870	
	30	#21 AND #29	17,259	(Intervention* [TIAB]) AND ((((((("post-traumatic stress disorder" [TIAB]) OR (PTSD [TIAB])) OR ("Acute Stress Disorder" [TIAB])) OR ("Psychological Distress" [TIAB])) OR ("Stress Disorders, Post-Traumatic" [MH])) OR ("Psychological Distress" [MH])) OR ("Stress Disorders, Traumatic, Acute" [MH]))

Search components	Search number	feed	Number of hits	Search string for MEDLINE via PubMed
Context	31	Hospital* [TIAB]	1,605,107	((((((Hospital* [TIAB]) OR ("acute hospital" [TIAB])) OR (clinic* [TIAB])) OR ("acute setting" [TIAB])) OR ("hospital setting" [TIAB])) OR (Hospitals [MH])) OR ("Subacute care" [MH])
	32	"acute hospital" [TIAB]	4,303	
	33	clinic* [TIAB]	5,160,437	
	34	"acute setting" [TIAB]	2,693	
	35	"hospital setting" [TIAB]	14,161	
	36	Hospitals [MH]	314,785	
	37	"Subacute care" [MH]	1,411	
	38	#31 OR #32 OR #33 OR #34 OR #35 OR #36 OR #37	6,264,657	
	39	COVID-19 [TIAB]	307,395	
	40	"COVID-19 pandemic" [TIAB]	115,894	
	41	COVID-19 [MH]	217,887	
	42	#39 OR #40 OR #41	335,265	((COVID-19 [TIAB]) OR ("COVID-19 pandemic" [TIAB])) OR (COVID-19 [MH])
	43	#20 AND #30 AND #38 AND #42	**192**	((((((((((((((((((((Physician* [TIAB]) OR (Doctor [TIAB])) OR ("Physician Assistant" [TIAB])) OR (Nurs* [TIAB])) OR ("Nursing staff" [TIAB])) OR (Caregiver* [TIAB])) OR ("Registered nurse" [TIAB])) OR ("Nurse practitioner" [TIAB])) OR ("Advanced practice nurse" [TIAB])) OR ("health care workers" [TIAB])) OR ("health workers" [TIAB])) OR ("health-care workers" [TIAB])) OR ("health care professionals" [TIAB])) OR ("frontline healthcare workers" [TIAB])) OR ("Nurse Clinicians" [MH])) OR ("Physician Assistants" [MH])) OR ("Nursing Staff, Hospital" [MH])) OR ("Health Personnel" [MH])) OR ("Nurse Specialists" [MH])) AND ((Intervention* [TIAB]) AND ((((((("post-traumatic stress disorder" [TIAB]) OR (PTSD [TIAB])) OR ("Acute Stress Disorder" [TIAB])) OR ("Psychological Distress" [TIAB])) OR ("Stress Disorders, Post-Traumatic" [MH])) OR ("Psychological Distress" [MH])) OR ("Stress Disorders, Traumatic, Acute" [MH])))) AND (((((((Hospital* [TIAB]) OR ("acute hospital" [TIAB])) OR (clinic* [TIAB])) OR ("acute setting" [TIAB])) OR ("hospital setting" [TIAB])) OR (Hospitals [MH])) OR ("Subacute care" [MH]))) AND (((COVID-19 [TIAB]) OR ("COVID-19 pandemic" [TIAB])) OR (COVID-19 [MH]))

Search component	Search number	Feed	Number of hits	Search string for MEDLINE via PubMed
	44	*#43 AND Filter: Humans, English, German*	**131**	((((((((((((((((((((Physician* [TIAB]) OR (Doctor [TIAB])) OR ("Physician Assistant" [TIAB])) OR (Nurs* [TIAB])) OR ("Nursing staff" [TIAB])) OR (Caregiver* [TIAB])) OR ("Registered nurse" [TIAB])) OR ("Nurse practitioner" [TIAB])) OR ("Advanced practice nurse" [TIAB])) OR ("health care workers" [TIAB])) OR ("health workers" [TIAB])) OR ("health-care workers" [TIAB])) OR ("health care professionals" [TIAB])) OR ("frontline healthcare workers" [TIAB])) OR ("Nurse Clinicians" [MH])) OR ("Physician Assistants" [MH])) OR ("Nursing Staff, Hospital" [MH])) OR ("Health Personnel" [MH])) OR ("Nurse Specialists" [MH])) AND ((Intervention* [TIAB]) AND (((((((("post-traumatic stress disorder" [TIAB]) OR (PTSD [TIAB])) OR ("Acute Stress Disorder" [TIAB])) OR ("Psychological Distress" [TIAB])) OR ("Stress Disorders, Post-Traumatic" [MH])) OR ("Psychological Distress" [MH])) OR ("Stress Disorders, Traumatic, Acute" [MH])))) AND (((((((Hospital* [TIAB]) OR ("acute hospital" [TIAB])) OR (clinic* [TIAB])) OR ("acute setting" [TIAB])) OR ("hospital setting" [TIAB])) OR (Hospitals [MH])) OR ("Subacute care" [MH]))) AND (((COVID-19 [TIAB]) OR ("COVID-19 pandemic" [TIAB])) OR (COVID-19 [MH])) Filters: English, German, Humans

Search in PsychINFO via EBSCO conducted at 16th April 2023 (search string is record online)

Table 10: Documentation of the search in PsychINFO via EBSCO (own representation)

Search component	Search number	Feed	Number of hits	Search string for PsychINFO via EBSCO
Population	1	TI Physician*	12,438	
	2	AB Physician*	67,087	
	3	TI Doctor	4,694	
	4	AB Doctor	29,205	
	5	TI “Doctor of medicine”	8	
	6	AB “Doctor of medicine”	55	
	7	TI “Physician Assistant”	113	
	8	AB “Physician Assistant”	278	
	9	TI Nurs*	47,624	
	10	AB Nurs*	109,210	
	11	TI “Nursing staff”	577	
	12	AB “Nursing staff”	3,688	
	13	TI Caregiver*	17,046	
	14	AB Caregiver*	57,076	
	15	TI “Registered nurse”	246	
	16	AB “Registered nurse”	1,134	
	17	TI “Nurse practitioner”	506	
	18	AB “Nurse practitioner”	1,260	
	19	TI "Advanced practice nurse”	53	
	20	AB “Advanced practice nurse”	231	
	21	TI “health care workers”	539	

Search component	Search number	Feed	Number of hits	Search string for PsychINFO via EBSCO
Population	22	AB “health care workers”	2,438	(TI Physician* OR AB Physician* OR TI Doctor OR AB Doctor OR TI “Doctor of medicine” OR AB “Doctor of medicine” OR TI “Physician Assistant” OR AB “Physician Assistant” OR TI Nurs* OR AB Nurs* OR TI “Nursing staff” OR AB “Nursing staff” OR TI Caregiver* OR AB Caregiver* OR TI “Registered nurse” OR AB “Registered nurse” OR TI “Nurse practitioner” OR AB “Nurse practitioner” OR TI “Advanced practice nurse” OR AB “Advanced practice nurse” OR TI “health care workers” OR AB “health care workers” OR TI “health workers” OR AB “health workers” OR TI “health-care workers” OR AB “health-care workers” OR TI “health care professionals” OR AB “health care professionals” OR DE Health Personnel OR DE Physicians OR DE Medical Personnel OR DE Clinicians OR DE Nurses OR DE Caregivers OR DE Professional Personnel OR DE Medical Personnel OR DE Frontline Employees)
	23	TI “health workers”	980	
	24	AB “health workers”	4,969	
	25	TI “health-care workers”	539	
	26	AB “health-care workers”	2,438	
	27	TI “health care professionals”	944	
	28	AB “health care professionals”	9,251	
	29	TI “frontline healthcare workers”	41	
	30	AB “frontline healthcare workers”	84	
	31	DE Health Personnel	26,167	
	32	DE Physicians	27,111	
	33	DE Medical Personnel	6,579	
	34	DE Clinicians	13,184	
	35	DE Nurses	33,523	
	36	DE Caregivers	41,950	
	37	DE Professional Personnel	5,534	
	38	DE Medical Personnel	6,579	
	39	DE Frontline Employees	208	
	40	#1 OR #2 OR #3 OR #4 Or #5 Or #6 Or #7 OR #8 Or #9 OR #10 OR #11 OR #12 OR #13 OR #14 Or #15 Or #16 Or #17 OR #18 OR #19 Or #20 OR #21 OR #22 OR #23 OR #24 OR #25 OR #26 OR #27 OR #28 OR #29 OR #30 OR #31 OR #32 OR #33 OR #34 OR #35 OR #36 OR #37 OR 38 OR #39	307,441	

Search component	Search number	Feed	Number of hits	Search string for PsychINFO via EBSCO
Concept	41	TI intervention*	86,159	(TI intervention* OR AB intervention* OR DE Intervention)
	42	AB intervention*	449,618	
	43	DE Intervention	86,100	
	44	#41 OR #42 OR #43	466,676	
	45	TI "post-traumatic stress disorder"	4,037	(TI "post-traumatic stress disorder" OR AB "post-traumatic stress disorder" OR TI PTSD OR AB PTSD OR TI "Acute Stress Disorder" OR AB "Acute Stress Disorder" OR TI "Psychological Distress" OR AB "Psychological Distress" OR DE "Posttraumatic Stress Disorder" OR DE "Posttraumatic Stress" OR DE Trauma OR DE "Acute Stress Disorder" OR DE "Stress and Trauma related disorders" OR DE "Caregiver burden")
	46	AB "post-traumatic stress disorder"	11,121	
	47	TI PTSD	8,833	
	48	AB PTSD	37,612	
	49	TI "Acute Stress Disorder"	270	
	50	AB "Acute Stress Disorder"	831	
	51	TI "Psychological Distress"	5,815	
	52	AB "Psychological Distress"	21,977	
	53	DE "Posttraumatic Stress Disorder"	39,317	
	54	DE "Posttraumatic Stress"	1,649	
	55	DE Trauma	28,905	
	56	DE "Acute Stress Disorder"	676	
	57	DE "Stress and Trauma related disorders"	48	
	58	DE "Caregiver burden"	7,090	
	59	#45 OR #46 OR #47 OR #48 OR #49 OR #50 OR #51 OR #52 OR #53 OR #54 OR #55 OR #56 OR #57 OR #58	**99,933**	

Search component	Search number	Feed	Number of hits	Search string for PsychINFO via EBSCO
	60	#44 AND #59	**20,547**	((TI intervention* OR AB intervention* OR DE intervention) AND (TI “post-traumatic stress disorder” OR AB “post-traumatic stress disorder” OR TI PTSD OR AB PTSD OR TI “Acute Stress Disorder” OR AB “Acute Stress Disorder” OR TI “Psychological Distress” OR AB “Psychological Distress” OR DE “Posttraumatic Stress Disorder” OR DE “Posttraumatic Stress” OR DE Trauma OR DE “Acute Stress Disorder” OR DE “Stress and Trauma related disorders” OR DE “Caregiver burden”))
**Context**	61	TI Hospital*	36,240	(TI Hospital* OR AB Hospital* OR TI “acute hospital” OR AB “acute hospital” OR TI clinic* OR AB clinic* OR TI “acute setting” OR AB “acute setting” OR TI “hospital setting” OR AB “hospital setting” OR DE Hospitals OR DE Clinics OR DE Treatment Facilities)
	62	AB Hospital*	169,250	
	63	TI “acute hospital”	223	
	64	AB “acute hospital”	830	
	65	TI clinic*	112,740	
	66	AB clinic*	675,499	
	67	TI “acute setting”	31	
	68	AB “acute setting”	263	
	69	TI “hospital setting”	676	
	70	AB “hospital setting”	2,632	
	71	DE Hospitals	19,101	
	72	DE Clinics	6,912	
	73	DE Treatment Facilities	2,132	
	74	#61 OR #62 OR #63 OR #64 OR #65 OR #66 OR #67 #OR #68 OR #69 OR #70 OR #71 OR #72 OR #73	**822,818**	
**Context**	78	TI COVID-19	21,000	(TI COVID-19 OR AB COVID-19 OR TI “COVID-19 pandemic” OR AB “COVID-19 pandemic” OR DE COVID-19)
	79	AB COVID-19	28,509	
	80	TI “COVID-19 pandemic”	7,952	
	81	AB “COVID-19 pandemic”	16,646	
	82	DE COVID-19	21,216	
	83	#78 OR #79 OR #80 OR #81 OR #82	**30,402**	

Search component	Search number	Feed	Number of hits	Search string for PsychINFO via EBSCO
	84	#40 AND #60 AND #74 AND #83	**83**	((TI Physician* OR AB Physician* OR TI Doctor OR AB Doctor OR TI "Doctor of medicine" OR AB "Doctor of medicine" OR TI "Physician Assistant" OR AB "Physician Assistant" OR TI Nurs* OR AB Nurs* OR TI "Nursing staff" OR AB "Nursing staff" OR TI Caregiver* OR AB Caregiver* OR TI "Registered nurse" OR AB "Registered nurse" OR TI "Nurse practitioner" OR AB "Nurse practitioner" OR TI "Advanced practice nurse" OR AB "Advanced practice nurse" OR TI "health care workers" OR AB "health care workers" OR TI "health workers" OR AB "health workers" OR TI "health-care workers" OR AB "health-care workers" OR TI "health care professionals" OR AB "health care professionals" OR DE Health Personnel OR DE Physicians OR DE Medical Personnel OR DE Clinicians OR DE Nurses OR DE Caregivers OR DE Professional Personnel OR DE Medical Personnel OR DE Frontline Employees) AND ((TI intervention* OR AB intervention* OR DE intervention) AND (TI "post-traumatic stress disorder" OR AB "post-traumatic stress disorder" OR TI PTSD OR AB PTSD OR TI "Acute Stress Disorder" OR AB "Acute Stress Disorder" OR TI "Psychological Distress" OR AB "Psychological Distress" OR DE "Posttraumatic Stress Disorder" OR DE "Posttraumatic Stress" OR DE Trauma OR DE "Acute Stress Disorder" OR DE "Stress and Trauma related disorders" OR DE "Caregiver burden")) AND (TI Hospital* OR AB Hospital* OR TI "acute hospital" OR AB "acute hospital" OR TI clinic* OR AB clinic* OR TI "acute setting" OR AB "acute setting" OR TI "hospital setting" OR AB "hospital setting" OR DE Hospitals OR DE Clinics OR DE Treatment Facilities) AND (TI COVID-19 OR AB COVID-19 OR TI "COVID-19 pandemic" OR AB "COVID-19 pandemic" OR DE COVID-19))
	85	AND *Filter: 2020-2023, Humans, English, German*	**83**	

9. Supplementary search options

This literature research is sensitive and aimed to identify preferably all possible references regarding the research question. Therefore Cooper et al. (9) conclude, that several supplementary search option should be conducted after the systematic research in databases.

This research focused on the topic of which interventions exist for symptoms of PTSD among hospitals-based nurses and physicians during the COVID-19 pandemic. First a limited systematic search is conducted in CINHAL via EBSCO, because this database contains literature specifically for the field literature from nursing science and related topics from the health care sector (see table 10). Furthermore, the publications exported to EndNote 20 and searched for duplicates before the additional title-abstract-screening is conducted in Rayyan (10). After that a total of two studies were included in full text screening (see supplement I).

Secondly, during the full text-screening of all included studies, the backwards citation tracking was conducted to search in the reference list of each included study for potential publications (e.g., study protocols) regarding the research question.

Thirdly, a search in study register (PROSEPRO, OSF, World Health Organization International Clinical Trials, EU Clinical Trials Register, German Register clinical trial, study register from U.S. National Institute of Health and ICTRN-study register for clinical trials in all fields of health care).

24th April 2023 the following findings resulted from this research in the study register from the U.S. National Institute of Health and ICTRN-study register for clinical trials in all fields of health care:

Table 11: Presentation of research findings from the supplementary search in study register (own visualization).

Title	Researchers	Duration	Content	Status
**Study register from the U.S. National Institute of Health**				
Reducing Posttraumatic Stress Disorder (PTSD) Symptoms in Frontline Health Care Workers	Garlick, James, Hemphill, Naomi	April 2023 until March 2026	Study address symptoms of PTSD in health care workers after the COVID-19 pandemic.<br>Hypothesis is, that the Prolonged Exposure for Primary Care (PE-PC)	Recruiting phase

			will reduce PTSD symptoms and improve functioning, compared to EAP Treatment as Usual (TAU).	
Health cAre woRkers exposed to COVID-19 (HARD COVID 19)	Responsible: University Hospital, Tours	From November 2020 until?	Evaluation of an intervention adapted to the exceptional circumstances of the crisis, like Depression, Burnout, Post Traumatic Stress Disorder. It´s designed as Trial(s) within Cohort design (TWIC). Intervention: Desensitization and neuro-emotional integration by eye movements (EMDR).	Active, not recruiting
Beyond Silence: Advancing E-mental Health Solutions to Support Canadian Healthcare Workers	Responsible: McMaster University, Canada	August 2022, estimated Study Completion: August 2023	Purpose is to scale implementation and evaluation of an m-Health app dsigned to promote early intervention and mental health support for frontline healthcare workers to reduce the risk of PTSD and/ or the mental health impact of the COVID-19 pandemic.	Not yet recruiting
Creative Arts Program to Reduce Burnout in Healthcare Professionals (CORAL)	Responsible: University of Colorado, Denver	March 2020 until March 2025	Question is, if creative arts programs that include visual, musical, written, or physical expression can reduce symptoms of burnout syndrome, Post Traumatic Stress Disorder (PTSD), depression, and anxiety in critical care healthcare professionals.	Recruiting
**ICTRN-study register for clinical trials in all fields of health care**				
Supporting healthcare workers with cognitive and behavioral coaching for posttraumatic	Oxford Centre for Anxiety Disorders and Trauma	October 2022 until March 2025	Intervention: SHAPE is a brief, accessible, remotely delivered intervention that targets cognitive processes and behaviors that maintain	Recruiting

stress disorder and depression.			PTSD and depression. Aim is to evaluate SHAPE. Participants: healthcare workers (e.g. doctors and nurses)	

Results from the backward citation tracking

Table 12: Presentation of results from the backward citation tracking (own visualization).

**Publication**	**Result**
Buselli et al. (2021)	Morina et al. (2021)<br>Mellins et al. (2020)<br>Albott et al. (2020)<br>Lefevre et al. (2021)
Hooper et al. (2021)	Blake et al. (2020)
Serrano-Ripoll (2020)	Fiol-DeRoque et al. (2021)

Other research options

Results from a systematic review by Hooper et al. (11) show, that some of the presented early psychological interventions were specifically developed to prevent or reduce symptoms of post-traumatic stress disorder (PTSD). For example, the 'Eye Movement and desensitization and reprocessing intervention' (EMDR) or the 'Psychological First Aid'(PFA).

In this case, the backwards citation tracking did not lead to any useful studies related to the research objectives. So, the researchers decided to conduct a web search via Google scholar with a combination of the following search terms:

- *name of the psychological intervention* mentioned in the systematic review by Hooper et al. (11)
- *PTSD*
- *health-care workers*
- *COVID-19*

For these psychological interventions, the following studies met the inclusion criteria:

Table 13: Results from a 'hand search' in Google scholar based on resulted psychological interventions from a systematic review by Hooper et al. (11) (own visualization).

Psychological First Aid (PFA)	Wang et al. (2022)

	Sulaiman et al. (2020)
Eye Movement Desensitization and Processing (EMDR)	Fogliato et al. (2022)<br>Sagaltici et al. (2022)

Search in CINAHL via EBSCO conducted at 18th April 2023 (search string is record online)

Table 14: Documentation of the search in CINAHL via EBSCO (own representation)

Search component	Search number	Feed	Number of hits	Search string for CINAHL via EBSCO
Population	1	TI Physician*	45,665	
	2	AB Physician*	146,113	
	3	TI Doctor	22,055	
	4	AB Doctor	49,049	
	5	TI “Doctor of medicine”	7	
	6	AB “Doctor of medicine”	212	
	7	TI “Physician Assistant”	1,085	
	8	AB “Physician Assistant”	1,272	
	9	TI Nurs*	368,585	
	10	AB Nurs*	387,874	
	11	TI “Nursing staff”	2,201	
	12	AB “Nursing staff”	11,345	
	13	TI Caregiver*	23,889	
	14	AB Caregiver*	54,817	
	15	TI “Registered nurse”	1,648	
	16	AB “Registered nurse”	4,886	
	17	TI “Nurse practitioner”	4,428	
	18	AB “Nurse practitioner”	5,606	
	19	TI "Advanced practice nurse”	469	
	20	AB “Advanced practice nurse”	879	
	21	TI “health care workers”	2,597	

Search component	Search number	Feed	Number of hits	Search string for CINAHL via EBSCO
	22	AB “health care workers”	6,691	(TI Physician* OR AB Physician* OR TI Doctor OR AB Doctor OR TI “Doctor of medicine” OR AB “Doctor of medicine” OR TI “Physician Assistant” OR AB “Physician Assistant” OR TI Nurs* OR AB Nurs* OR TI “Nursing staff” OR AB “Nursing staff” OR TI Caregiver* OR AB Caregiver* OR TI “Registered nurse” OR AB “Registered nurse” OR TI “Nurse practitioner” OR AB “Nurse practitioner” OR TI “Advanced practice nurse” OR AB “Advanced practice nurse” OR TI “health care workers” OR AB “health care workers” OR TI “health workers” OR AB “health workers” OR TI “health-care workers” OR AB “health-care workers” OR TI “health care professionals” OR AB “health care professionals” OR TI “frontline healthcare workers” OR AB “frontline healthcare workers” OR MH Physicians OR MH “Physician Assistants” OR MH Nurses OR MH "Practical Nurses" OR MH "Nursing Staff, Hospital" OR MH "Staff Nurses" OR MH Caregivers OR MH "Registered Nurses" OR MH "Practical Nurses" OR MH "Nurse Practitioners" OR MH "Advanced Practice Nurses" OR MH "Health Personnel")
	23	TI “health workers”	2,735	
	24	AB “health workers”	9,058	
	25	TI “health-care workers”	2,597	
	26	AB “health-care workers”	6,691	
	27	TI “health care professionals”	2,308	
	28	AB “health care professionals”	17,584	
	29	TI “frontline healthcare workers”	50	
	30	AB “frontline healthcare workers”	143	
	31	MH Physicians	66,776	
	32	MH "Physician Assistants"	5,675	
	33	MH Nurses	69,672	
	34	MH "Practical Nurses"	5,857	
	35	MH "Nursing Staff, Hospital"	24,553	
	36	MH "Staff Nurses"	8,213	
	37	MH Caregivers	42,752	
	38	MH "Registered Nurses"	36,065	
	39	MH "Practical Nurses"	5,857	
	40	MH "Nurse Practitioners"	20,139	
	41	MH "Advanced Practice Nurses"	1,989	
	42	MH "Health Personnel"	51,350	

	43	#1 OR #2 OR #3 OR #4 Or #5 Or #6 Or #7 OR #8 Or #9 OR #10 OR #11 OR #12 OR #13 OR #14 Or #15 Or #16 Or #17 OR #18 OR #19 Or #20 OR #21 OR #22 OR #23 OR #24 OR #25 OR #26 OR #27 OR #28 OR #29 OR #30 OR #31 OR #32 OR #33 OR #34 OR #35 OR #36 OR #37 OR 38 OR #39 OR #40 OR #41 OR #42	**997,988**	

Search component	Search number	Feed	Number of hits	Search string for CINAHL via EBSCO
Concept of interest	44	TI intervention*	118,385	(TI intervention* OR AB intervention*)
	45	AB intervention*	504,651	
	46	#44 OR #45	551,649	
	47	TI “post-traumatic stress disorder”	2,250	(TI “post-traumatic stress disorder” OR AB “post-traumatic stress disorder” OR TI PTSD OR AB PTSD OR TI “Acute Stress Disorder” OR AB “Acute Stress Disorder” OR TI “Psychological Distress” OR AB “Psychological Distress” OR MH "Stress Disorders, Post-Traumatic" OR MH "Psychological Distress")
	48	AB “post-traumatic stress disorder”	5,845	
	49	TI PTSD	4,332	
	50	AB PTSD	12,387	
	51	TI “Acute Stress Disorder”	124	
	52	AB “Acute Stress Disorder”	288	
	53	TI “Psychological Distress”	4,224	
	54	AB “Psychological Distress”	13,142	
	55	MH "Stress Disorders, Post-Traumatic"	26,951	
	56	MH "Psychological Distress"	4,783	
	57	#47 OR #48 OR #49 OR #50 OR #51 OR #52 OR #53 OR #54 OR #55 OR #56 OR	**47,113**	
	58	#46 AND #57	**9,678**	

Search component	Search number	Feed	Number of hits	Search string for CINAHL via EBSCO
Context	59	TI Hospital*	161,045	
	60	AB Hospital*	478,899	
	61	TI “acute hospital”	872	
	62	AB “acute hospital”	2,677	
	63	TI clinic*	339,738	
	64	AB clinic*	1,135,481	
	65	TI “acute setting”	169	
	66	AB “acute setting”	966	
	67	TI “hospital setting”	1,876	
	68	AB “hospital setting”	5,872	
	69	MH Hospitals	67,009	
	70	OR #59 OR #60 OR #61 OR #62 OR #63 OR #64 OR #65 OR #66 OR #67 #OR #68 OR #69	**1,715,286**	(TI Hospital* OR AB Hospital* OR TI “acute hospital” OR AB “acute hospital” OR TI clinic* OR AB clinic* OR TI “acute setting” OR AB “acute setting” OR TI “hospital setting” OR AB “hospital setting” OR MH Hospitals)
Context	71	TI COVID-19	84,312	
	72	AB COVID-19	69,800	
	73	TI “COVID-19 pandemic”	20,575	
	74	AB “COVID-19 pandemic”	31,900	
	75	MH „COVID-19“	40,295	
	76	#71 OR #72 OR #73 OR #74 OR #75	**115,144**	(TI COVID-19 OR AB COVID-19 OR TI “COVID-19 pandemic” OR AB “COVID-19 pandemic” OR MH “COVID-19”)
	77	#43 AND #58 AND #70 AND #76	**61**	
	78	AND *Filter: 2020-2023, Humans, English, German*	**59**	

10. Title-Abstract- and full-text-screening

All included references were export to EndNote 20 and after a screening for duplicates, the references were transferred to Rayyan (10). There, the title-abstract-, as well as the full-text-screening took place by the first author herself.

To establish methodological quality, four studies were independently reviewed MR and DH, each. On 18th April 2023 the results were discussed and one reference, which was set on "maybe" by DK was included after a discussion.

From 19th until 21thApril 2023 the full-text-screening was conducted also in Rayyan by DK and three references were screened by MR and DH, independently.

The Discussion took place on 4th April 2023, as well as the alignment with the MR and DH.

11.References

1. Peters MDJ, Marnie C, Tricco AC, Pollock D, Munn Z, Alexander L, et al. Updated methodological guidance for the conduct of scoping reviews. JBI Evid Synth 2020;18(10):2119–26. PubMed PMID: 33038124.
2. Couarraze S, Delamarre L, Marhar F, Quach B, Jiao J, Avilés Dorlhiac R, et al. The major worldwide stress of healthcare professionals during the first wave of the COVID-19 pandemic - the international COVISTRESS survey. PloS one 2021;16(10):e0257840. PubMed PMID: 34614016; PubMed Central PMCID: PMC8494302.
3. Saragih ID, Tonapa SI, Saragih IS, Advani S, Batubara SO, Suarilah I, et al. Global prevalence of mental health problems among healthcare workers during the Covid-19 pandemic: A systematic review and meta-analysis. Int J Nurs Stud 2021;121:104002. PubMed PMID: 34271460; PubMed Central PMCID: PMC9701545.
4. Stuijfzand S, Deforges C, Sandoz V, Sajin C-T, Jaques C, Elmers J, et al. Psychological impact of an epidemic/pandemic on the mental health of healthcare professionals: a rapid review. BMC Public Health 2020;20(1):1230. PubMed PMID: 32787815; PubMed Central PMCID: PMC7422454.
5. Evanoff BA, Strickland JR, Dale AM, Hayibor L, Page E, Duncan JG, et al. Work-Related and Personal Factors Associated With Mental Well-Being During the COVID-19 Response: Survey of Health Care and Other Workers. J Med Internet Res 2020;22(8):e21366. PubMed PMID: 32763891; PubMed Central PMCID: PMC7470175.
6. McGowan J, Sampson M, Salzwedel DM, Cogo E, Foerster V, Lefebvre C. PRESS Peer Review of Electronic Search Strategies: 2015 Guideline Statement. J Clin Epidemiol 2016;75:40–6. PubMed PMID: 27005575.
7. Nordhausen T, Hirt J. RefHunter im neuen Webformat: Eine Plattform zur systematischen Literaturrecherche. GMS Medizin - Bibliothek - Information 2022;22 de.
8. Liang L, Gao T, Ren H, Cao R, Qin Z, Hu Y, et al. Post-traumatic stress disorder and psychological distress in Chinese youths following the COVID-19 emergency.

J Health Psychol 2020;25(9):1164–75. PubMed PMID: 32627606; PubMed Central PMCID: PMC7342938.

9. Cooper C, Booth A, Britten N, Garside R. A comparison of results of empirical studies of supplementary search techniques and recommendations in review methodology handbooks: a methodological review. Syst Rev 2017;6(1):234. PubMed PMID: 29179733; PubMed Central PMCID: PMC5704629.
10. Ouzzani M, Hammady H, Fedorowicz Z, Elmagarmid A. Rayyan-a web and mobile app for systematic reviews. Syst Rev 2016;5(1):210. PubMed PMID: 27919275; PubMed Central PMCID: PMC5139140.
11. Hooper JJ, Saulsman L, Hall T, Waters F. Addressing the psychological impact of COVID-19 on healthcare workers: learning from a systematic review of early interventions for frontline responders. BMJ Open 2021;11(5):e044134. PubMed PMID: 34020974; PubMed Central PMCID: PMC8142676.
12. Page MJ, McKenzie JE, Bossuyt PM, Boutron I, Hoffmann TC, Mulrow CD, et al. The PRISMA 2020 statement: an updated guideline for reporting systematic reviews. BMJ 2021;372:n71. PubMed PMID: 33782057; PubMed Central PMCID: PMC8005924.

12.Supplement I Flowchart

(I)

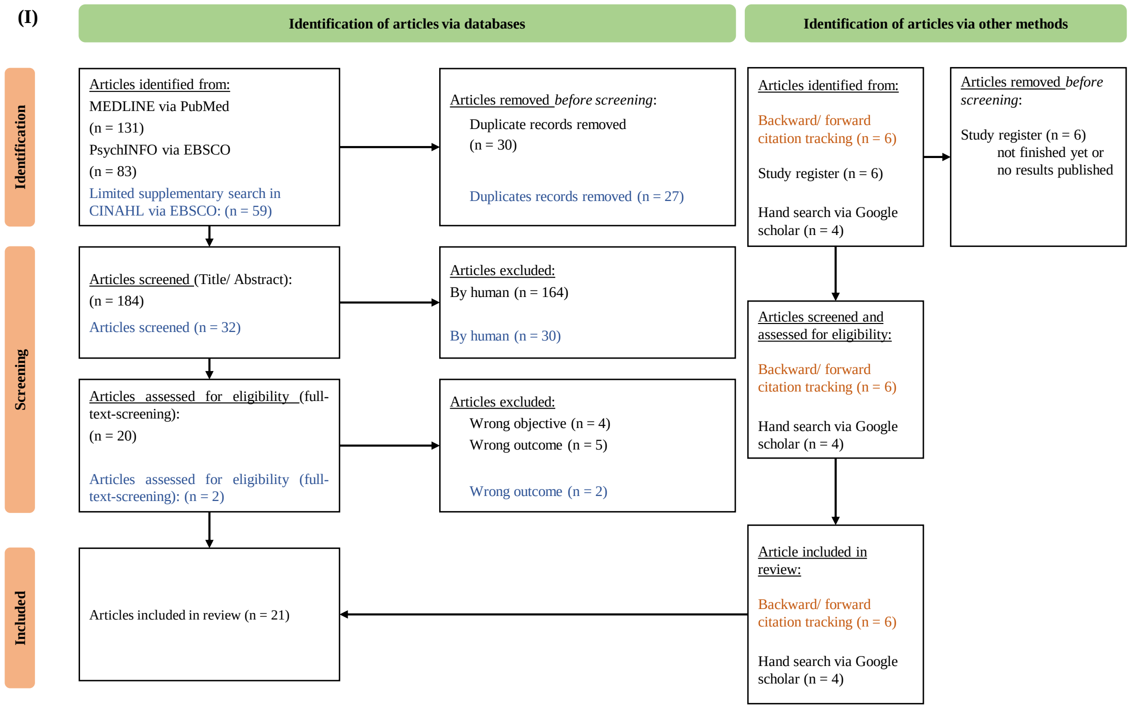

(12)

13 Supplement II Bibliographic information of the articles included for data extraction

(1) Albott, C. S., Wozniak, J. R., McGlinch, B. P., Wall, M. H., Gold, B. S., & Vinogradov, S. (2020). Battle Buddies: Rapid Deployment of a Psychological Resilience Intervention for Health Care Workers During the COVID-19 Pandemic. *Anesth Analg*, *131*(1), 43-54. https://doi.org/10.1213/ANE.0000000000004912

(2) Blake, H., Bermingham, F., Johnson, G., & Tabner, A. (2020). Mitigating the Psychological Impact of COVID-19 on Healthcare Workers: A Digital Learning Package. *Int J Environ Res Public Health*, *17*(9). https://doi.org/10.3390/ijerph17092997

(3) Bureau, R., Bemmouna, D., Faria, C. G. F., Goethals, A.-A. C., Douhet, F., Mengin, A. C., Fritsch, A., Zinetti Bertschy, A., Frey, I., & Weiner, L. (2021). My health too: Investigating the feasibility and the acceptability of an internet-based cognitive-behavioral therapy program developed for healthcare workers. *Frontiers in Psychology*, *12*. https://doi.org/10.3389/fpsyg.2021.760678

(4) Dong, L., Meredith, L. S., Farmer, C. M., Ahluwalia, S. C., Chen, P. G., Bouskill, K., Han, B., Qureshi, N., Dalton, S., Watson, P., Schnurr, P. P., Davis, K., Tobin, J. N., Cassells, A., & Gidengil, C. A. (2022). Protecting the mental and physical well-being of frontline health care workers during COVID-19: Study protocol of a cluster randomized controlled trial. *Contemp Clin Trials*, *117*, 106768. https://doi.org/10.1016/j.cct.2022.106768

(5) Dumarkaite, A., Truskauskaite, I., Andersson, G., Jovarauskaite, L., Jovaisiene, I., Nomeikaite, A., & Kazlauskas, E. (2023). The efficacy of the internet-based stress recovery intervention FOREST for nurses amid the COVID-19 pandemic: A randomized controlled trial. Int J Nurs Stud, 138, 104408. https://doi.org/doi:10.1016/j.ijnurstu.2022.104408

(6) Fiol-DeRoque, M. A., Serrano-Ripoll, M. J., Jimenez, R., Zamanillo-Campos, R., Yanez-Juan, A. M., Bennasar-Veny, M., Leiva, A., Gervilla, E., Garcia-Buades, M. E., Garcia-Toro, M., Alonso-Coello, P., Pastor-Moreno, G., Ruiz-Perez, I., Sitges, C., Garcia-Campayo, J., Llobera-Canaves, J., & Ricci-Cabello, I. (2021). A Mobile Phone-Based Intervention to Reduce Mental Health Problems in Health Care Workers During the COVID-19 Pandemic (PsyCovidApp): Randomized

Controlled Trial. JMIR Mhealth Uhealth, 9(5), e27039. https://doi.org/10.2196/27039

(7) Fogliato, E., Invernizzi, R., Maslovaric, G., Fernandez, I., Rigamonti, V., Lora, A., Frisone, E., & Pagani, M. (2022). Promoting Mental Health in Healthcare Workers in Hospitals Through Psychological Group Support With Eye Movement Desensitization and Reprocessing During COVID-19 Pandemic: An Observational Study. *Front Psychol*, *12*, 794178. https://doi.org/10.3389/fpsyg.2021.794178

(8) Hannig, C., Lotzin, A., Milin, S., & Schäfer, I. (2021). Stress- und Traumaprävention für Beschäftigte im Gesundheitsbereich = Stress and trauma prevention for employees in the health sector. *Trauma & Gewalt*, *15*(3), 232-242. https://doi.org/10.21706/tg-15-3-232

(9) Jovarauskaite, L., Dumarkaite, A., Truskauskaite-Kuneviciene, I., Jovaisiene, I., Andersson, G., & Kazlauskas, E. (2021). Internet-based stress recovery intervention FOREST for healthcare staff amid COVID-19 pandemic: study protocol for a randomized controlled trial. *Trials*, *22*(1), 559. https://doi.org/10.1186/s13063-021-05512-1

(10) Kanellopoulos, D., Solomonov, N., Ritholtz, S., Wilkins, V., Goldman, R., Schier, M., Oberlin, L., Bueno-Castellano, C., Dargis, M., Cherestal, S., & Gunning, F. (2021). The CopeNYP program: A model for brief treatment of psychological distress among healthcare workers and hospital staff. *Gen Hosp Psychiatry*, *73*, 24-29. https://doi.org/10.1016/j.genhosppsych.2021.09.002

(11) Lefevre, H., Stheneur, C., Cardin, C., Fourcade, L., Fourmaux, C., Tordjman, E., Touati, M., Voisard, F., Minassian, S., Chaste, P., Moro, M. R., & Lachal, J. (2021). The Bulle: Support and Prevention of Psychological Decompensation of Health Care Workers During the Trauma of the COVID-19 Epidemic. *J Pain Symptom Manage*, *61*(2), 416-422. https://doi.org/10.1016/j.jpainsymman.2020.09.023

(12) Mellins, C. A., Mayer, L. E. S., Glasofer, D. R., Devlin, M. J., Albano, A. M., Nash, S. S., Engle, E., Cullen, C., Ng, W. Y. K., Allmann, A. E., Fitelson, E. M., Vieira, A., Remien, R. H., Malone, P., Wainberg, M. L., & Baptista-Neto, L. (2020). Supporting the well-being of health care providers during the COVID-19 pandemic: The CopeColumbia response. *Gen Hosp Psychiatry*, *67*, 62-69. https://doi.org/10.1016/j.genhosppsych.2020.08.013

(13) Morina, N., Weilenmann, S., Dawson, K. S., Ernst, J., Zanitti, Z., von Känel, R., Schick, M., Spiller, T. R., & Bryant, R. A. (2021). RECHARGE - A Brief Psychological Intervention to Build Resilience in Health Care Workers During the COVID-19 Pandemic: Study Protocol for a Randomized Controlled Trial *Preprint* https://doi.org/10.21203/rs.3.rs-212942/v1

(14) Sagaltici, E., Saydam, R. B., Cetinkaya, M., Şahin Ş, K., Küçük, S. H., & Müslümanoğlu, A. Y. (2022). Burnout and psychological symptoms in healthcare workers during the COVID-19 pandemic: Comparisons of different medical professions in a regional hospital in Turkey. *Work*, *72*(3), 1077-1085. https://doi.org/10.3233/wor-210517

(15) Serrano-Ripoll, M. J., Ricci-Cabello, I., Jiménez, R., Zamanillo-Campos, R., Yañez-Juan, A. M., Bennasar-Veny, M., Sitges, C., Gervilla, E., Leiva, A., García-Campayo, J., García-Buades, M. E., García-Toro, M., Pastor-Moreno, G., Ruiz-Perez, I., Alonso-Coello, P., Llobera, J., & Fiol-deRoque, M. A. (2021). Effect of a mobile-based intervention on mental health in frontline healthcare workers against COVID-19: Protocol for a randomized controlled trial. *J Adv Nurs*, *77*(6), 2898-2907. https://doi.org/10.1111/jan.14813

(16) Solomonov, N., Kanellopoulos, D., Grosenick, L., Wilkins, V., Goldman, R., Ritholtz, S., Falk, A., & Gunning, F. M. (2022). CopeNYP: A brief remote psychological intervention reduces health care workers' depression and anxiety symptoms during COVID-19 pandemic. *World Psychiatry*, *21*(1), 155-156. https://doi.org/10.1002/wps.20946

(17) Sulaiman, A. H., Ahmad Sabki, Z., Jaafa, M. J., Francis, B., Razali, K. A., Juares Rizal, A., Mokhtar, N. H., Juhari, J. A., Zainal, S., & Ng, C. G. (2020). Development of a Remote Psychological First Aid Protocol for Healthcare Workers Following the COVID-19 Pandemic in a University Teaching Hospital, Malaysia. *Healthcare (Basel)*, *8*(3). https://doi.org/10.3390/healthcare8030228 (Sulaiman et al., 2020)

(18) Trottier, K., Monson, C. M., Kaysen, D., Wagner, A. C., Liebman, R. E., & Abbey, S. E. (2022). Initial findings on RESTORE for healthcare workers: an internet-delivered intervention for COVID-19-related mental health symptoms. *Transl Psychiatry*, *12*(1), 222. https://doi.org/10.1038/s41398-022-01965-3

(19) Trottier, K., Monson, C. M., Kaysen, D., Wagner, A. C., Pun, C., & Abbey, S. E. (2021). Development of RESTORE: an online intervention to

improve mental health symptoms associated with COVID-19-related traumatic and extreme stressors. *Eur J Psychotraumatol*, *12*(1), 1984049. https://doi.org/doi:10.1080/20008198.2021.1984049

(20) Wang, L., Norman, I., Xiao, T., Li, Y., Li, X., & Leamy, M. (2022). Evaluating a Psychological First Aid Training Intervention (Preparing Me) to Support the Mental Health and Wellbeing of Chinese Healthcare Workers During Healthcare Emergencies: Protocol for a Randomized Controlled Feasibility Trial. *Front Psychiatry*, *12*, 809679. https://doi.org/10.3389/fpsyt.2021.809679

(21) Weiner, L., Berna, F., Nourry, N., Severac, F., Vidailhet, P., & Mengin, A. C. (2020). Efficacy of an online cognitive behavioral therapy program developed for healthcare workers during the COVID-19 pandemic: the REduction of STress (REST) study protocol for a randomized controlled trial. *Trials*, *21*(1), 870. https://doi.org/10.1186/s13063-020-04772-7
